# Mild and selective reduction of aldehydes utilising sodium dithionite under flow conditions

**DOI:** 10.3762/bjoc.14.129

**Published:** 2018-06-22

**Authors:** Nicole C Neyt, Darren L Riley

**Affiliations:** 1 Department of Chemistry, University of Pretoria, Pretoria 0002, South Africa

**Keywords:** aldehyde reduction, flow chemistry, selective reduction, sodium dithionite

## Abstract

We recently reported a novel hybrid batch–flow synthesis of the antipsychotic drug clozapine in which the reduction of a nitroaryl group is described under flow conditions using sodium dithionite. We now report the expansion of this method to include the reduction of aldehydes. The method developed affords yields which are comparable to those under batch conditions, has a reduced reaction time and improved space-time productivity. Furthermore, the approach allows the selective reduction of aldehydes in the presence of ketones and has been demonstrated as a continuous process.

## Introduction

Flow chemistry and continuous processing has been acknowledged by the American Chemical Society (ACS), the Green Chemistry Institute (GCI) and several global pharmaceutical companies as one of the primary areas for research and development for chemical manufacturing [1–4]. For the past decade flow chemistry and the application of flow devices has been gaining acceptance in laboratories because of its ease of use, safety and control [3–6]. Continuous flow technologies are generally more effective than traditional batch processes with key advantages including intensified heat and mass transfer, inline reaction monitoring, higher mass throughput, safer control of hazardous chemicals increasing lab safety and direct scalability. These are all beneficial in moving towards more efficient and sustainable techniques in chemical processing [3–4,6–7].

The reduction of carbonyl groups are a standard type of transformation in organic synthesis, however, to date under flow conditions reductions have mostly been limited to soluble reducing agents like DIBAL [7–10] and superhydride [11] which require special handling or expensive solid-supported borohydride species [12]. Recently, Seeberger and co-workers demonstrated a more cost-effective sodium borohydride-mediated flow reduction utilizing solid mixes of sodium borohydride, lithium chloride and celite [12], and the Ley group were able to demonstrate a green transfer hydrogenation of ketones under flow using catalytic lithium *tert*-butoxide in isopropanol [13].

**Scheme 1:**
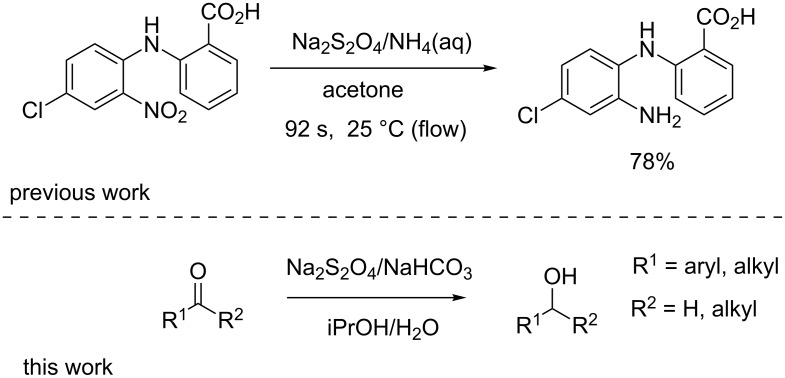
Sodium dithionite-mediated reductions under basic conditions.

We recently published a batch–flow hybrid synthesis of the antipsychotic drug clozapine in which we demonstrated a flow-based reduction of a nitro group utilising sodium dithionite as a reductant [14]. We further hypothesized that the development of a flow protocol for the reduction of carbonyl groups would also be possible using sodium dithionite (Scheme 1) [15–16].

Sodium dithionite can act as a powerful reducing agent [16], is mild, easy to use, and it is compatible with protic solvents like water and isopropanol. It has previously been used to reduce a range of different organic functional groups including aldehydes and ketones [17–18], pyridinium ions to afford piperidines [17], benzil groups [19], nitroarenes and nitroalkanes in the presence of dialkyl viologen electron transfer catalysts [20–21] and immobilized nitroarene’s under phase transfer conditions [22–23].

In this publication we report the efficient reduction of aldehydes under flow conditions utilising sodium dithionite under basic conditions and the expansion of the approach to allow the selective reduction of aldehydes in the presence of ketones.

## Results and Discussion

### Batch-based reduction of aldehydes and ketones

The reduction of simple aldehydes and ketones (Table 1, Scheme 1) were envisaged and successfully demonstrated utilising sodium dithionite under standard batch conditions by refluxing for 12 hours in the presence of sodium dithionite and sodium bicarbonate (1 M) in an isopropanol/water mixture. Several primary and secondary alcohols (Table 1) were prepared in yields of 52–98% and 49–73%, respectively.

**Table 1:** Reduction of aldehydes and ketones under batch and flow conditions.

entry	R^1^	R^2^	yield^a,b^		productivity^c^		flow productivity/ batch productivity

			batch^a^	flow^b^	batch (g·L^−1^·h^−1^)	flow (g·L^−1^·h^−1^)	

1.1	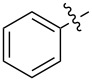	H	92%	92%	0.96	4.27	4.4
1.2	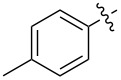	H	80%	81%	0.84	4.24	5.0
1.3	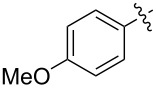	H	89%	73%	0.93	4.32	4.6
1.4	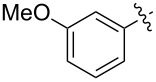	H	83%	77%	0.87	4.55	5.2
1.5	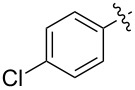	H	98%	70%	1.02	4.30	4.2
1.6	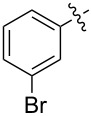	H	69%	80%	0.72	2.01	2.8
1.7	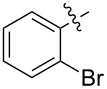	H	85%	88%	0.88	7.05	8.0
1.8	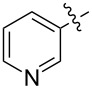	H	92%	80%	0.96	3.75	3.9
1.9	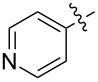	H	79%	91%	0.83	1.45	1.7
1.10	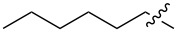	H	65%	71%	0.68	3.54	5.2
1.11	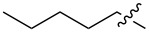	H	52%	68%	0.55	2.97	5.4
1.12	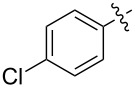	Me	58%	11%^d^	0.61	0.72	1.2
1.13	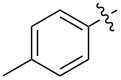	Me	60%	4%^d^	0.63	0.23	0.4
1.14	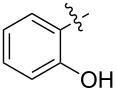	Me	50%	<1%^d^	0.52	<0.08	0.1
1.15	cyclohexanone		84%	50%^d^	0.88	2.16	2.4
1.16	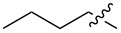	Et	49%	<1%^d^	0.51	<0.05	0.1
1.17	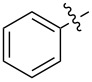	Me	73%	29%^d^	0.76	1.51	2.0

^a^Batch conditions: aldehyde or ketone (1 equiv), Na_2_S_2_O_4_ (4.5 equiv), NaHCO_3_/IPA (1:1), Δ, 12 h. ^b^Flow conditions: for aldehydes, 0.165 M aldehyde (1 equiv) in IPA/H_2_O (1:1), 0.75 M Na_2_S_2_O_4_ (4.5 equiv) in NaHCO_3_/IPA (1:1), 0.250 mL·min^−1^ (64 min residence time for entries 1.1–1.5, 1.7, 1.8, 1.10, and 1.11) or 0.1 mL·min^−1^ (160 min residence time for entries 1.6 and 1.9), 110 °C; for ketones, 0.165 M ketone (1 equiv) in IPA/H_2_O (1:1), 0.75 M Na_2_S_2_O_4_ (4.5 equiv) in NaHCO_3_/IPA (1:1), 0.200 mL·min^−1^ (80 min residence time). ^c^Productivity (space-time) = grams of product produced per L reactor volume per hour. Batch reactions performed on 1 g scale, reactor volume = 81 mL, reaction time = 12 h, flow reactions performed on 1.65 mmol scale (64 min for entries 1.1–1.5, 1.7, 1.8, 1.10, and 1.11) or 0.99 mmol scale (160 min for entries 1.6 and 1.9), reactor volume = 16 mL (2 mL chip + 14 mL coil), reaction time = 2.4 h (64 min residence) or 4.67 h (160 min residence) [24]. ^d^Conversions estimated from ^1^H NMR.

### Flow-based reduction of aldehydes and ketones

**Figure 1:**
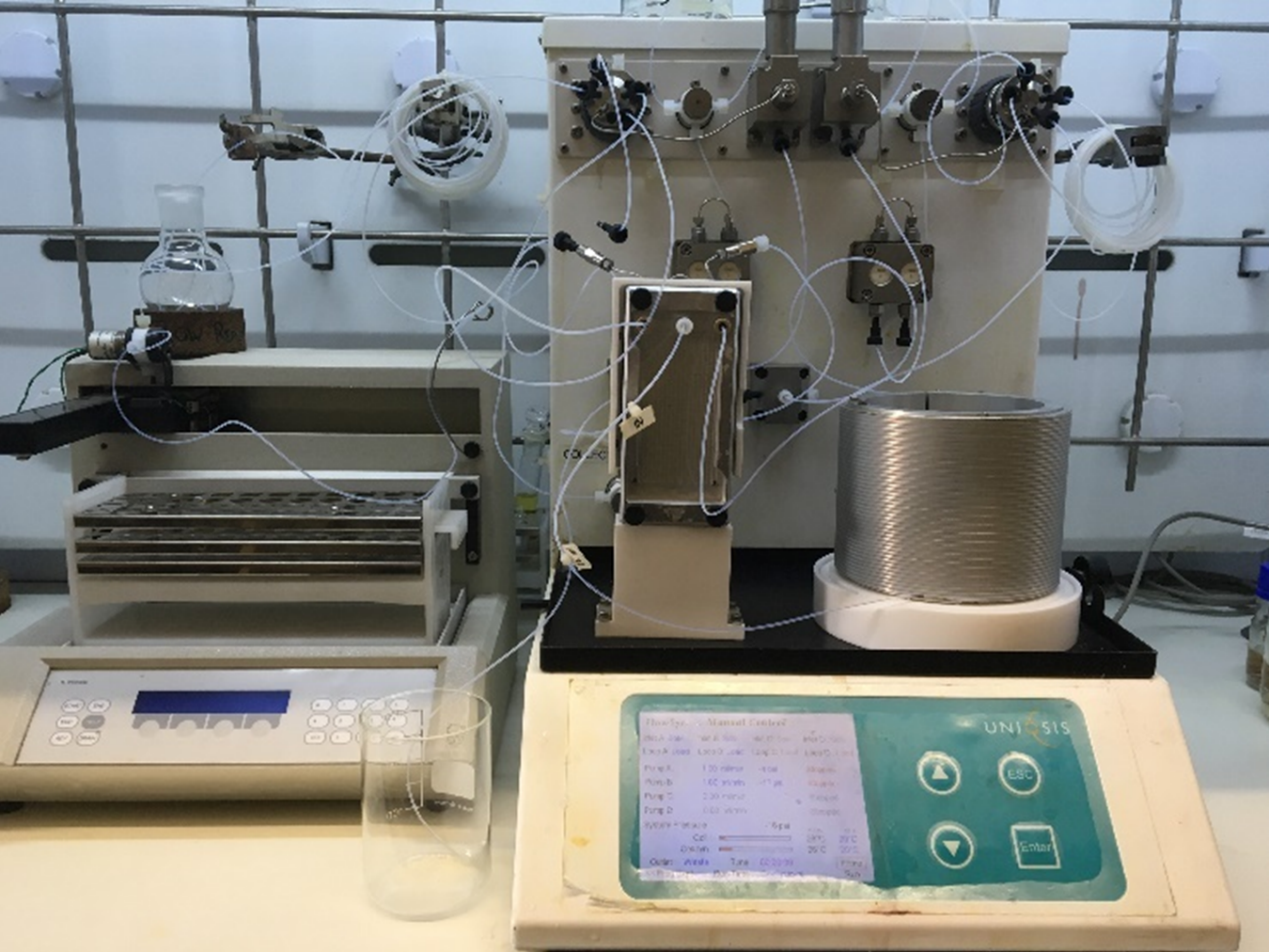
Uniqsis FlowSyn Stainless Steel Flow reactor.

**Figure 2:**
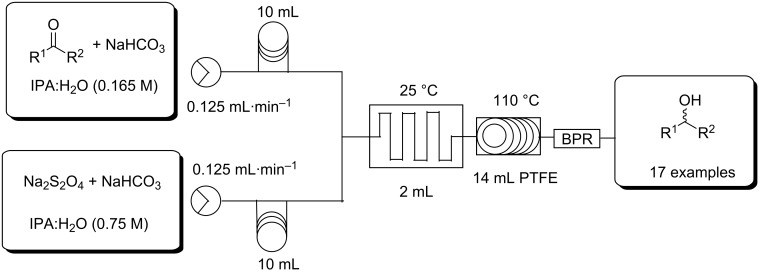
Flow reactor configuration for the reduction of aldehydes and ketones.

In developing a flow protocol a Uniqsis FlowSyn Stainless Steel Flow reactor with a Multi X automated sampler was utilized. The reactor set-up (Figure 1) involved the use of two HPLC pumps, a 2 mL mixing chip connected in series to a 14 mL HT PTFE coil mounted on a heating block and a back pressure regulator fitted at the output flow stream. Reagents were introduced through two 10 mL injection loops (Figure 2).

### Optimisation under flow conditions

**Figure 3:**
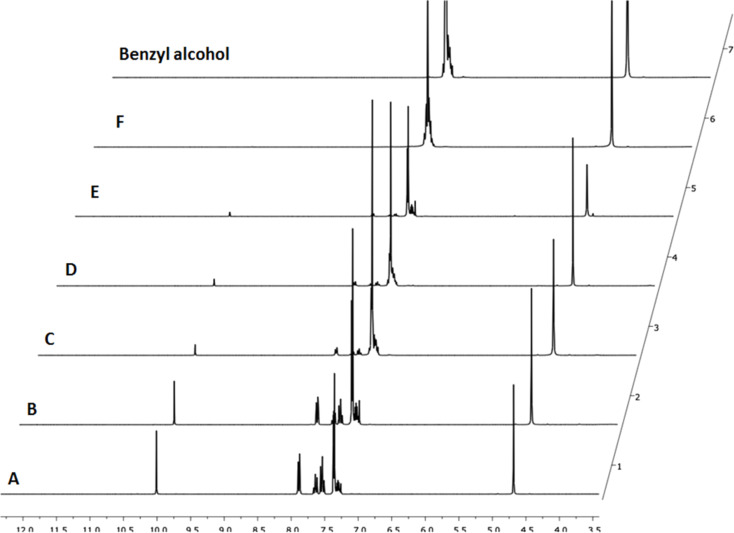
NMR spectra showing the optimisation of the dithionite reduction for the reduction of benzaldehyde.

The flow reduction was initially optimised for the conversion of benzaldehyde to benzyl alcohol. The process required 4.5 equivalents of sodium dithionite and was optimised in terms of flow rate and temperature using the reactor set-up shown in Figure 2. Under optimised conditions 0.75 M sodium dithionite in isopropanol/water/NaHCO_3_ [1 M] (1:1:2) was mixed with a 0.165 M stock solution of benzaldehyde in isopropanol/water/NaHCO_3_ [1 M] (1:1:2) in a 2 mL mixing chip at ambient temperature. Thereafter, the solution was superheated to 110 °C while being passed through a 14 mL HT PTFE coil affording near quantitative conversion (92% isolated yield) with a residence time of 64 minutes in the mixing chip and heated coil reactor (Table 2, Figure 3). In order to better compare the flow and batch processes the reduction of benzaldehyde was repeated in a seal-tube vessel superheated to 110 °C with a reaction time of 64 min to match that of the optimised flow process. In this instance a 43% conversion to benzyl alcohol was observed, indicating that under flow conditions, the simple superheating of the solvent was only partly responsible for the increased rate of reaction and that in all likelihood the improved mixing of the reagent streams also plays an important role.

**Table 2:** Optimization of the reduction of benzaldehyde under flow conditions.

entry	residence time (min)	flow rate (mL·min^–1^)	temp (°C)	conversion	yield

A	64	0.25	50	51%	–
B	64	0.25	70	70%	–
C	64	0.25	90	89%	–
D	53	0.30	110	90%	–
E	59	0.27	110	92%	–
F	64	0.25	110	99%	92%

Further reduction of the residence time while maintaining quantitative conversion was envisaged by increasing the reaction temperature beyond 110 °C, however, the deposition of a black residue within the reactor eventually led to blockages and reactor fouling. The residue is possibly arising from the decomposition of sodium thiosulfate which occurs when heating in aqueous solutions above 90 °C. The precipitate was not observed in the reactor at temperatures up to 110 °C and as such the flow process was limited to a maximum operating temperature of 110 °C.

The optimised conditions were translated to the reduction of the remaining aldehydes (Table 1). Under these conditions, aldehyde reductions gave moderate to good yields (68–92%) which were comparable to those observed in batch in a significantly reduced reaction residence time (64 min vs 12 h). When expressed in terms of reactor space-time productivity (grams of product produced per litre of reactor volume per hour) the flow based processes displayed productivities which ranged from 3.9 to 8 times that observed under batch conditions for the runs with a residence time of 64 min (Table 1, entries 1.1–1.5, 1.7, 1.8, 1.11, and 1.12). It should be noted that the batch-based processes were not stringently optimised in terms of reaction residence time with reactions being stopped and worked-up after 12 h, however, at this point in time only the reduction of benzaldehyde and 4-chlorobenzaldehyde (Table 1, entries 1.1 and 1.5) showed complete consumption of starting material. In the case of a few selected examples (Table 1, entries 1.6 and 1.9) a longer residence time of 160 min was investigated, however, the decrease in reactor productivity on increasing the reaction residence time meant that the advantage afforded by the flow approach decreased with productivity differences of <1.9 times relative to the corresponding batch processes.

When the approach was adopted for the reduction of ketones, surprisingly low conversions of <50% were observed when residence times ranged from 80 to 160 min, with only the reduction of cyclohexanone affording a moderate yield of 50%. In all cases the yields were considered to be too low to be of use even though in certain examples (Table 1, entries 1.15 and 1.17) the productivity was arguably moderately better (2.4 and 2.0 times, respectively) than that of the analogous batch processes.

### Selective reduction of aldehydes in the presence of ketones

The striking difference in relative reactivity between the ketones and the aldehydes on the flow system allowed us to demonstrate selective reductions of aldehydes in the presence of various ketones by simple selection of an appropriate flow rate. This selectivity was shown by the reduction of benzaldehyde in the presence of various ketones at equal concentrations (Table 3). In all cases benzaldehyde was efficiently reduced (71–91% conversion as determined by ^1^H NMR) and the ketones remained largely unreduced with only acetophenone (9%) and 4-chloroacetophenone (8%) affording conversions above 1% (Table 3, entries 3.1 and 3.2).

**Table 3:** Selective reduction of benzaldehyde in the presence of various ketones.

entry	ketone^a^	aldehyde reduction	ketone reduction

3.1	acetophenone	80%	≤9%
3.2	4-chloroacetophenone	91%	8%
3.3	4-methylacetophenone	72%	≤1%
3.4	2-hydroxyacetophenone	72%	≤1%
3.5	4-aminoacetophenone	85%	≤1%
3.6	cyclohexanone	71%	≤1%
3.7	3-heptanone	72%	≤1%
3.8	3-acetylbenzaldehyde	85%	≤1%

^a^Stock solution of 0.2 M concentration relative to both benzaldehyde and ketone in IPA/H_2_O/NaHCO_3_ (1:1:2) [1 M], 0.9 M sodium dithionite (4.5 equiv) in IPA/H_2_O/NaHCO_3_ (1:1:2) [1 M], flow rate 0.250 mL·min^−1^ (64 min residence in mixing chip and coil reactor), 110 °C; ^b^0.165 M of substrate in IPA/H_2_O/NaHCO_3_ (1:1:2) [1 M], 0.75 M sodium dithionite (4.5 equiv) in IPA/H_2_O/NaHCO_3_ (1:1:2) [1 M], flow rate 0.250 mL·min^−1^, 110 °C.

**Figure 4:**
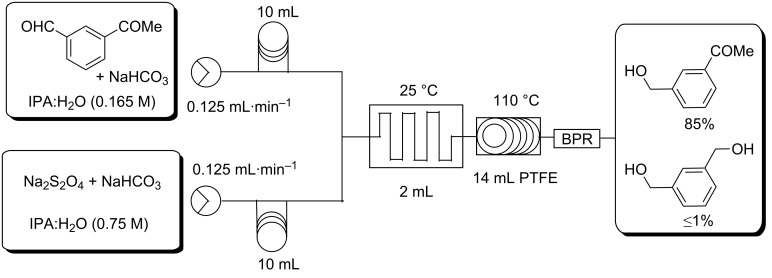
Selective reduction of an aldehyde in the presence of a ketone.

The reaction was further tested by the reduction of 3-acetylbenzaldehyde which contained an aldehyde and ketone functionality on the same molecular scaffold. In this instance an aldehyde conversion of 85% was observed with <1% reduction of the ketone (Table 3, entry 3.8, Figure 4).

### Demonstration of a continuous process

**Figure 5:**
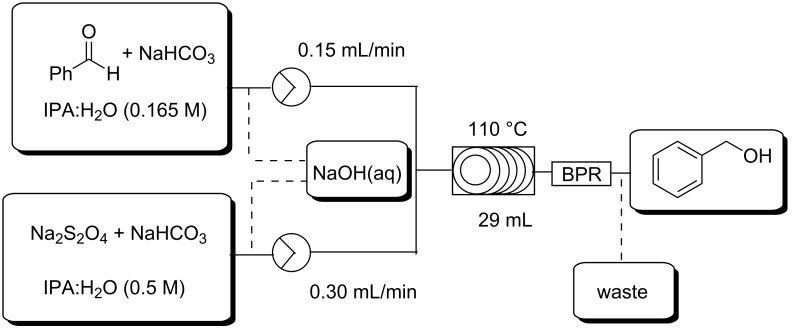
Flow reactor set-up for the continuous reduction of aldehydes.

Finally, we were interested in converting the approach to a continuously run process, however, a concern at this stage was the formation of the aforementioned precipitate and although this was negligible at 110 °C, we felt it could potentially lead to blockages and reactor fouling over extended reaction times. In consideration of this we modified the reactor set-up illustrated in Figure 1 by removing the mixing chip which previously acted as a trap for precipitates and diluting the sodium dithionite stock solution from 0.75 M to 0.5 M (Figure 5). As a safety precaution a stream of aqueous sodium hydroxide was connected to the two reagent streams via selector valves which could be used to flush the reactor if blockages occurred. Finally the injection loops were exchanged for reagent reservoirs and the 14 mL coil reactor was exchanged for a 29 mL coil reactor (Figure 5). The process was tested with a 64 min residence time (0.45 mL·min^−1^ flow rate) and allowed to run continuously for 55.3 hours during which time no significant precipitate formation was noted and no reactor flushing was required. A total of 8.7 g of benzaldehyde was reduced affording 6.99 g of benzylalcohol (79% yield after chromatographic purification) equating to a productivity of 4.36 g·L^−1^·h^−1^ which was comparable to that of 4.27 g·L^−1^·h^−1^ observed for the reduction of benzaldehyde using the setup shown in Figure 2 (Table 1, entry 1.1).

## Conclusion

In conclusion, we have developed a simple transition-metal-free continuous flow method for the reduction of aldehydes in aqueous media utilising sodium dithionite which does not generate or use molecular hydrogen. The process affords comparable yields to those obtained under batch conditions but in reduced reaction residence time (64 min vs 12 h) and improved (>3.9 times) space-time productivity. The process shows high relative selectivity for the reduction of aldehydes over ketones and through the appropriate selection of flow rate selective reductions of aldehydes in the presence of ketones can be realised. Finally the process can be run continuously with minimal loss in reactor productivity.

## Supporting Information

File 1 Experimental part.
