# Supplementary material for: Mild and selective reduction of aldehydes utilising sodium dithionite under flow conditions
Source: Beilstein J Org Chem. 2018 Jun 22;14:1529–36. doi: 10.3762/bjoc.14.129 (PMC6036987; doi:10.3762/bjoc.14.129)
Supplement: File 1 — Experimental part. [file Beilstein_J_Org_Chem-14-1529-s001.pdf]

# Supporting Information

**for**

# Mild and selective reduction of aldehydes utilising sodium dithionite under flow conditions

Nicole C. Neyt and Darren L. Riley*

Address: Department of Chemistry, University of Pretoria, Pretoria 0002, South Africa

Email: Darren L. Riley - darren.riley@up.ac.za

* Corresponding author

## Experimental part

Table of Contents

Experimental ........ S3
General methods ........ S3
General method for the reduction of aldehydes and ketones under traditional batch conditions ........ S3
General method for the reduction of aldehydes and ketones under flow conditions ........ S3
Reduction of benzaldehyde in a sealed tube ........ S4
General method for the selective reduction of aldehydes in the presence of ketones ........ S4
Method for scaled up reduction of benzaldehyde ........ S4
References ........ S8
$^{1}$H NMR and $^{13}$C NMR spectra of reduced aldehydes: ........ S9
Phenylmethanol ........ S9
(4-Methylphenyl)methanol ........ S10
(4-Methoxyphenyl)methanol ........ S11
(4-Chlorophenyl)methanol ........ S13

(3-Bromophenyl)methanol ........................................................................................ S14
(2-Bromophenyl)methanol ........................................................................................ S15
Pyridin-3-ylmethanol ............................................................................................... S16
Heptanol.................................................................................................................. S17
Hexanol ................................................................................................................... S18
1-(4-Chlorophenyl)ethanol and 4-chloroacetophenone mixture .............................................. S19
1-(*p*-Tolylethanol) and 4-methylacetophenone mixture ....................................................... S20
2-(1-Hydroxyethyl)phenol and 2-hydroxyacetophenone mixture............................................. S21
............................................................................................................................. S21
Cyclohexanol ........................................................................................................... S22
$^{1}$H NMR for reduced ketones in flow: ............................................................................ S25
1-(4-Chlorophenyl)ethanol and 4-chloroacetophenone mixture .............................................. S25
1-(4-Methylphenyl)ethanol and 4-methylacetophenone mixture ............................................ S25
1-(2-Hydroxyphenyl)ethanol and 2-hydroxyacetophenone mixture......................................... S26
Cyclohexanol and cyclohexanone mixture ................................................................... S26
3-Heptanone (no 3-heptanol formation)..................................................................... S27
1-Phenylethanol and acetophenone mixture ............................................................... S27
............................................................................................................................. S27
$^{1}$H NMR and $^{13}$C NMR spectrum of reduced benzaldehyde in the presence of a ketone:.......... S28
Benzaldehyde and acetophenone mixture .................................................................. S28
3-Acetylbenzaldehyde and 1-(3-(hydroxymethyl)phenyl)ethenone mixture ...................... S30

## Experimental

## General methods

All solvents, chemicals, and reagents were obtained commercially and used without further purification. $^1$H NMR (300 MHz) and $^{13}$C NMR (75 MHz) spectra were recorded on Bruker AVANCE-III-300 instrument using $CDCl_3$ or DMSO-$d_6$ as solvent. $CDCl_3$ contained tetramethylsilane as an internal standard. Chemical shifts, δ, are reported in parts per million (ppm), and splitting patterns are given as singlet (s), doublet (d), triplet (t), quartet (q), or multiplet (m). Coupling constants, *J,* are expressed in hertz (Hz). In noted cases conversions were determined from NMR by comparison of integral areas of starting materials and products. Infrared spectra were run on a Bruker ALPHA Platinum ATR spectrometer. The absorptions are reported on the wavenumber (cm$^{-1}$) scale, in the range 400–4000 cm$^{-1}$. The retention factor ($R_f$) values quoted are for thin-layer chromatography (TLC) on aluminium-backed Macherey-Nagel ALUGRAM Sil G/UV254 plates pre-coated with 0.25 mm silica gel 60, spots were visualised with UV light and basic $KMnO_4$ spray reagent. Chromatographic separations were performed on Macherey-Nagel Silica gel 60 (particle size 0.063–0.200 mm). Yields refer to isolated pure products unless stated otherwise.

## General method for the reduction of aldehydes and ketones under traditional batch conditions

Benzaldehyde (1 g, 9.5 mmol, 1 equiv) was dissolved in 38 mL (1:1 IPA/ $H_2O$), (0.25 M). Sodium dithionite (7.5 g, 43 mmol, 4.5 equiv) and $NaHCO_3$ (1.6 g, 19 mmol, 2 equiv) was dissolved in water (43 mL, [1 M]) and added to the aldehyde. The mixture was refluxed for 12 hours under argon. The solution was allowed to cool to room temperature and the products were extracted using EtOAc (3 × 50 mL). This was dried using $Na_2SO_4$, filtered and dried under vacuum with a yield of 0.95 g (92%). For entry 1.15 the compound was neutralized with 1 M HCl and extracted with EtOAc (3 × 50 mL) and washed with water (3 × 50 mL) the organic extracts were combined and dried using $Na_2SO_4$. The solvent was evaporated *in vacuo* and the resulting residue purified using column chromatography. Unless specified a 3:1 EtOAc/hexane eluent was used for chromatographic purification [1-3].

## General method for the reduction of aldehydes and ketones under flow conditions

A 0.165 M stock solution of aldehyde or ketone (1 equiv) was prepared by dissolution in (1:1:2 IPA/$H_2O$/$NaHCO_3$ [1 M]). A 0.75 M stock solution of sodium dithionite (4,5 equiv) was prepared by dissolution in (1:1:2 IPA/$H_2O$/$NaHCO_3$ [1 M]). The injection loops (10 mL) unless otherwise stated

were primed with the two stock solutions respectively. The solutions were then pumped continuously through a 2 mL mixing chip at ambient temperature followed by a 14 mL HT Teflon coil at 110 °C (aldehydes and ketones). Unless otherwise stated the flow rate was set to 0.25 mL·min$^{-1}$ (64 min residence) for aldehydes and 0.20 mL·min$^{-1}$ (80 min residence) for ketones. Product work-up and isolation was achieved using the approach described for the batch reductions.

## Reduction of benzaldehyde in a sealed tube

## General method for the selective reduction of aldehydes in the presence of ketones

A stock solution of 0.2 M concentration with respect to both the ketone and benzaldehyde was prepared by dissolution in (1:1:2 IPA/$H_2O$/$NaHCO_3$ [1 M]). A stock solution of 0.9 M sodium dithionite (4.5 equiv) was prepared by dissolutionin (1:1:2 IPA/$H_2O$/$NaHCO_3$ [1 M]). The injection loops (10 mL) were primed with the two stock solutions respectively. The solutions were pumped continuously at a flow rate of 0.25 mL·min$^{-1}$ through a 2 mL mixing chip at ambient temperature followed by a 14 mL HT Teflon coil heated to 110 °C. Product isolation was not performed, and conversion was estimated from $^1$H NMR

## Method for scaled up reduction of benzaldehyde

A 0.165 M stock solution of benzaldehyde (A) (1 equiv) was prepared by dissolution in (1:1:2 IPA/$H_2O$/$NaHCO_3$ [1 M]). A 0.5 M stock solution of sodium dithionite (B) (4.5 equiv) was prepared by dissolution in (1:1:2 IPA/$H_2O$/$NaHCO_3$ [1 M]). The inlet bottles were charged with each stock solution A and B respectively. Pump A was set to 0.15 mL·min$^{-1}$ and pump B set to a flow rate of 0.3 mL·min$^{-1}$. The solutions were pumped continuously through a T-piece at ambient temperature followed by a 29 mL coil (14 mL HT Teflon coil and 15 mL stainless steel coil) at 110 °C. The reaction was halted after 55 hours and 18 minutes. Thereafter, the flow reactor was washed with 1 M NaOH to remove any precipitate. The reaction mixture was extracted with EtOAc (5 × 100 mL). The organic extracts were combined and dried using $Na_2SO_4$. The solvent was evaporated, and the crude material obtained was purified by column chromatography 3:1 EtOAc/hexane eluent was used for chromatographic purification. to afford 6.99 g phenylmethanol in 79% yield.

**Phenylmethanol (1.1)**: Colorless oil, batch yield 937 mg (92%); isolated flow yield 164 mg (92%). Rf 0.78 (3:1 EtOAc/Hexanes); $^1$H NMR (300 MHz, $CDCl_3$) δ 7.48–7.18 (m, 5H), 4.65 (s, 2H), 2.33 (s, 1H); $^{13}$C NMR (75MHz, $CDCl_3$) δ 140.80, 128.46, 127.53, 126.92, 65.12; IR $v_{max}$/cm$^{-1}$ 3311, 3030, 2871, 1493, 1451, 1204, 1078, 1010, 910, 734 and 693. In agreement with Sigma Aldrich database https://www.sigmaaldrich.com/spectra/fnmr/FNMR009403.PDF.

**4-Methylbenzyl alcohol (1.2)**: White solid, batch yield 82 mg (80%, reaction performed on 100 mg scale), isolated flow yield 163 mg (81%); mp 57 - 60°C; Rf 0.65 (3:1 EtOAc/Hexanes); $^{1}$H NMR (300 MHz, $CDCl_3$) δ 7.26 (d, *J* = 7.9 Hz, 2H), 7.17 (d, *J* = 7.9 Hz, 2H), 4.64 (s, 2H), 2.36 (s, 3H), 1.81 (s, 1H); $^{13}$C NMR (75 MHz, $CDCl_3$) δ 137.88, 137.35, 129.20,127.08, 65.20, 21.13; IR $v_{max}/cm^{-1}$ 3345, 2917, 2856, 1675, 1608, 1573, 1513, 1443, 1412, 1343, 1280, 1207, 1177, 1115, 1010. In agreement with Sigma Aldrich database https://www.sigmaaldrich.com/spectra/fnmr/FNMR000756.PDF.

**4-Methoxybenzyl alcohol (1.3)**: Colorless oil, batch yield 907 mg (89%), isolated flow yield 166 mg (73%); Rf 0.56(3:1 EtOAc/Hexanes); $^{1}$H NMR (300 MHz, $CDCl_3$) δ 7.28 (d, *J* = 8.6 Hz, 2H), 6.89 (d, *J* = 8.6 Hz, 2H), 4.60 (m, 2H), 3.80 (s, 3H), 1.78 (s, 1H); $^{13}$C NMR (75 MHz, $CDCl_3$) δ 159.19, , 133.14, 128.60, 113.94, 64.96, 55.29; IR $v_{max}/cm^{-1}$ 3423, 2936, 2837, 1608, 1509, 1460, 1299, 1240, 1171, 1028, 813 and 756. In agreement with Sigma Aldrich database https://www.sigmaaldrich.com/spectra/fnmr/FNMR001049.PDF.

**3-Methoxybenzyl alcohol (1.4)**: Colorless oil, batch yield 844 mg (83%), isolated flow yield 175 mg (77%); Rf 0.51(3:1 EtOAc/Hexanes); $^{1}$H NMR (300 MHz, $CDCl_3$) δ 7.27 (t, *J* = 8.1 Hz, 1H), 6.93 (d, *J* = 7.1 Hz, 2H), 6.84 (d, *J* = 9.0 Hz, 1H), 4.66 (s, 2H), 3.81 (s, 3H), 1.89 (s, 1H); $^{13}$C NMR (75 MHz, $CDCl_3$) δ 159.81, 142.52, 129.57, 119.07, 113.24, 112.22, 65.21, 55.17; IR $v_{max}/cm^{-1}$ 3333, 2937, 2837, 1592, 1487, 1258, 1153, 1033, 858, 779, 734 and 690. In agreement with Sigma Aldrich database https://www.sigmaaldrich.com/spectra/fnmr/FNMR010797.PDF.

**4-Chlorobenzyl alcohol (1.5)**: White solid, batch yield 995 mg (98%), isolated flow yield 0.165 mg (70%); mp 73-75°C; Rf 0.68 (3:1 EtOAc/Hexanes); $^{1}$H NMR (300 MHz, $CDCl_3$) δ 7.32 – 7.25 (m, 2H), 7.25 – 7.19 (m, 2H), 4.65 (s, 2H), 1.88 (s, 1H); $^{13}$C NMR (75 MHz, $CDCl_3$) δ 139.26, 133.35, 128.66, 128.24, 64.51; IR $v_{max}/cm^{-1}$ 3325, 2920, 2855, 1902, 1486, 1450, 1401, 1344, 1204, 1083, 1002, 828 and 647. In agreement with Sigma Aldrich database https://www.sigmaaldrich.com/spectra/fnmr/FNMR009685.PDF.

**3-Bromobenzyl alcohol (1.6):** Colourless oil, batch yield 697 mg (69%), isolated flow yield (6 mL reaction loops) 150 mg (80%), Rf 0.79 (3:1 EtOAc/Hexanes);$^{1}$H NMR (300 MHz, $CDCl_3$) δ 7.54 (s, 1H), 7.44 (dd, *J* = 7.5, 1.9 Hz, 1H), 7.26 (q, *J* = 7.5 Hz, 1H), 4.68 (s, 1H), 2.35 (s, 1H);$^{13}$C NMR (75 MHz, $CDCl_3$) δ 143.05, 130.63, 130.10, 129.86, 125.30, 122.63, 64.48; IR $v_{max}/cm^{-1}$3302, 2924, 2872, 1693, 1596, 1569, 1468, 1425, 1196, 1016, 881, 837, 774, 667. In agreement with Sigma Aldrich database https://www.sigmaaldrich.com/spectra/fnmr/FNMR002447.PDF.

**2-Bromobenzyl alcohol (1.7)**: White solid, batch yield 859 mg (85%), isolated flow yield 271 mg (88%); mp 75 - 76°C; Rf 0.84 (3:1 EtOAc/Hexanes); 1H NMR (300 MHz, $CDCl_3$) δ 7.54 (d, *J* = 9.1 Hz,

1H), 7.48 (d, $J$ = 6.0 Hz, 1H), 7.33 (dd, $J$ = 7.5 Hz, 1H), 7.16 (dd, $J$ = 7.7 Hz, 1H), 4.74 (s, 2H), 2.13 (s, 1H); $^{13}$C NMR (75 MHz, $CDCl_3$) δ 139.69, 132.57, 129.09, 128.87, 127.63, 122.54, 65.04; IR $v_{max}/cm^{-1}$ 3197, 2907, 2852, 1466, 1433, 1359, 1190, 1049, 1015, 741 and 660.

**Pyridin-3-ylmethanol (1.8)**: Colorless oil, batch yield 937 mg (92%), isolated flow yield 144 mg (80%); Rf 0.10 (3:1 EtOAc/Hexanes); $^1$H NMR (300 MHz, $CDCl_3$) δ8.51 – 8.31 (m, 2H), 7.72 (d, $J$ = 7.8 Hz, 1H), 7.26 (dd, $J$ = 7.6 & 5.0 Hz, 1H), 4.68 (s, 2H), 4.40 (s, 1H); $^{13}$C NMR (75 MHz, $CDCl_3$) δ 147.90, 147.74, 137.15, 135.37, 123.64, 62.04; IR $v_{max}/cm^{-1}$ 3207, 2917, 2854, 1584, 1425, 1216, 1101, 1021, 788, 707, 635.

**Pyridin-3-ylmethanol (1.9):** White solid, batch yield 330 mg (79%), isolated flow yield (6 mL reaction loops) 108 mg (91%); mp 51 - 55$^o$C, Rf 0.2 (3:1 EtOAc/Hexanes);$^1$H NMR (400 MHz, $CDCl_3$) δ 8.40 (d, $J$ = 3.6 Hz, 2H), 7.29 (d, $J$ = 4.9 Hz, 2H), 4.72 (s, 2H), 4.81 (s, 1H); $^{13}$C NMR (101 MHz, $CDCl_3$) δ 151.66, 148.98, 121.32, 62.72; IR $v_{max}/cm^{-1}$ 3116, 2904, 2855, 2818, 1603, 1561, 1455, 1414, 1296, 1225, 1094, 1053, 999, 795, 732.

**Heptanol (1.10)**: Colorless oil, batch yield 657 mg (65%), isolated flow yield 136 mg (71%); Rf 0.62 (1:3 EtOAc/Hexanes); $^1$H NMR (300 MHz, $CDCl_3$) δ3.62 (t, $J$ = 6.6 Hz, 2H), 1.65 – 1.48 (m, 3H), 1.38 – 1.21 (m, 8H), 0.93 – 0.81 (m, 3H); $^{13}$C NMR (75MHz, $CDCl_3$) δ63.01, 32.76, 31.79, 29.07, 25.67, 22.57, 14.03; IR $v_{max}/cm^{-1}$ 3320, 2924, 2857, 1460, 1376, 1054, 722. In agreement with Sigma Aldrich database https://www.sigmaaldrich.com/spectra/fnmr/FNMR010620.PDF.

**Hexanol (1.11)**: Colorless oil, batch yield 530 mg (52%), isolated flow yield 114 mg (68%); Rf 0.89 (3:1 EtOAc/Hexanes); $^1$H NMR (300 MHz, $CDCl_3$) δ3.63 (t, $J$ = 6.6 Hz, 2H), 1.78 (s, 1H), 1.65 – 1.46 (m, 2H), 1.42 – 1.18 (m, 4H), 1.00 – 0.78 (m, 3H);$^{13}$C NMR (75 MHz, $CDCl_3$) δ 63.03, 32.73, 31.61, 25.39, 22.60, 13.99; IR $v_{max}/cm^{-1}$ 3340, 2926, 2860, 1460, 1377, 1187, 1050, 921 and 725. In agreement with Sigma Aldrich database

**1-(4-Chlorophenyl)ethanol (1.12)**: Colorless oil, batch conversion 58%, flow conversion 11%; Rf 0.68 (3:1 EtOAc/Hexanes); $^1$H NMR (300 MHz, $CDCl_3$) δ 7.27 (s, 4H), 4.83 (q, $J$ = 6.5 Hz, 1H), 2.47 (s, 1H), 1.44 (d, $J$ = 6.5 Hz, 3H); $^{13}$C NMR (75 MHz, $CDCl_3$) δ 129.74, 128.89, 128.53, 126.81, 69.61, 25.24. IR $v_{max}/cm^{-1}$ 3340 (C-OH); **4-chloroacetophenone**: $^1$H NMR (300 MHz, $CDCl_3$) δ 7.92 – 7.79 (m, 2H), 7.45 – 7.36 (m, 2H), 2.55 (s, 3H); $^{13}$C NMR (75 MHz, $CDCl_3$) δ 197.09, 144.36, 139.61, 135.36, 132.94, 26.54; IR $v_{max}/cm^{-1}$ 1682(C=O). In agreement with Sigma Aldrich database https://www.sigmaaldrich.com/spectra/fnmr/FNMR001389.PDF.

**1-(4-Methylphenyl)ethanol (1.13)**: Colorless oil batch conversion 60% flow conversion 4%; Rf 0.66 (3:1 EtOAc/Hexanes); $^1$H NMR (300 MHz, $CDCl_3$) δ 7.26 (m, 4H), 4.85 (q, $J$ = 6.4 Hz, 1H), 2.35 (s, 3H),

2.26 (s, 1H), 1.48 (d, *J* = 6.5 Hz, 3H); $^{13}$C NMR (75 MHz, $CDCl_3$) δ 129.26, 129.12, 128.47, 125.38, 70.14, 26.52, 25.13;IR $v_{max}$/$cm^{-1}$ 3336 (C-OH); **4-methylacetophenone**:$^{1}$H NMR (300 MHz, $CDCl_3$) δ 7.86 (d, *J* = 8.2 Hz, 1H), 7.15 (d, *J* = 7.9 Hz, 1H), 2.57 (s, 3H), 2.42 (s, 3H); $^{13}$C NMR (75 MHz, $CDCl_3$) δ 198.04, 143.93, 137.01, 134.72, 21.65, 21.11;IR $v_{max}$/$cm^{-1}$ 1677(C=O)

**1-(2-Hydroxyphenyl)ethanol (1.14)**: Colorless oil, batch conversion 50%, flow conversion <1%; Rf 0.37 (3:1 EtOAc/Hexanes); $^{1}$H NMR (300 MHz, $CDCl_3$) δ 8.16 (s, 1H), 7.19 – 7.11 (m, 1H), 6.98 (dd, *J* = 7.4 & 1.5 Hz, 1H), 6.85 (dd, *J* = 7.8 & 2.4 Hz, 2H), 5.04 (q, *J* = 6.6 Hz, 1H), 3.30 (s, 1H), 1.56 (d, *J* = 6.6 Hz, 3H); $^{13}$C NMR (75MHz, $CDCl_3$) δ 155.22, 128.77, 128.57, 126.41, 119.82, 116.90, 71.18, 23.32; IR $v_{max}$/$cm^{-1}$ 3390 (C-OH); **2-hydroxyacetophenone**: $^{1}$H NMR (300 MHz, $CDCl_3$) δ12.26 (s, 1H), 7.71 (dd, *J* = 8.0, 1.6 Hz, 1H), 7.44 (ddd, *J* = 8.6, 7.4, 1.6 Hz, 1H), 6.95 (dd, *J* = 8.4, 0.8 Hz, 1H), 6.91 – 6.84 (m, 1H), 2.60 (s, 3H);$^{13}$C NMR (75 MHz, $CDCl_3$) δ 204.46, 162.23, 136.52, 130.72,119.57, 118.97, 118.33, 26.48; IR $v_{max}$/$cm^{-1}$ 1636 (C=O)

**Cyclohexanol (1.15)**: Colorless oil, batch conversion 84%, flow conversion 50%; Rf 0.46 (1:3 EtOAc/Hexanes); $^{1}$H NMR (300 MHz, $CDCl_3$) δ 3.61 (dq, *J* = 13.1 & 4.3 Hz, 1H), 1.97 – 1.80 (m, 2H), 1.80 – 1.64 (m, 2H), 1.61 – 1.45 (m, 2H), 1.37 – 1.10 (m, 5H); $^{13}$C NMR (75 MHz, $CDCl_3$) δ 70.33, 35.53, 25.44, 24.11; IR $v_{max}$/$cm^{-1}$ 3326, 2926, 2853, 1449, 1359, 1293, 1260, 1230, 1137, 1065, 1025, 965, 888, 841, 786.

**3-Heptanol (1.16)**: Colorless oil, batch conversion 49%, flow conversion ≤1%; Rf 0.46 (1:3 EtOAc/Hexanes); $^{1}$H NMR (300 MHz, $CDCl_3$)δ 3.52 – 3.40 (m, 1H), 1.76 (s, 1H) 1.46 – 1.33 (m, 4H), 1.33 – 1.24 (m, 4H), 0.88 (t, *J* = 7.5 Hz, 6H); $^{13}$C NMR (75 MHz, $CDCl_3$) δ 73.09, 36.53, 30.02, 27.75, 22.66, 13.93, 9.74; IR $v_{max}$/$cm^{-1}$ 3490 (C-OH); **3-heptanone**: $^{1}$H NMR (300 MHz, $CDCl_3$) δ 2.50 – 2.21 (m, 4H), 1.50 (dt, *J* = 15.3 & 7.4 Hz, 2H), 1.37 – 1.09 (m, 2H), 0.99 (t, *J* = 7.3 Hz, 3H), 0.84 (t, *J* = 7.3 Hz, 3H). $^{13}$C NMR (75 MHz, $CDCl_3$) δ 211.86, 42.00, 35.71, 25.92, 22.25, 13.71, 7.69; IR $v_{max}$/$cm^{-1}$ 1711(C=O). In agreement with Sigma Aldrich database https://www.sigmaaldrich.com/spectra/fnmr/FNMR000240.PDF.

**1-Phenylethanol (1.17)**: Colorless oil, batch conversion 73%, flow conversion 29% (flow rate 0.2 $ml.min^{-1}$) or 73% (flow rate 0.075 $ml.min^{-1}$); Rf 0.80 (3:1 EtOAc/Hexanes); $^{1}$H NMR (300 MHz, $CDCl_3$) δ 7.40 – 7.22 (m, 5H), 4.86 (q, *J* = 6.5 Hz, 1H), 1.48 (d, *J* = 6.5 Hz, 3H); $^{13}$C NMR (75 MHz, $CDCl_3$) δ 128.44, 128.30, 127.22, 125.28, 70.10, 25.04. IR $v_{max}$/$cm^{-1}$ 3372 (C-OH); **acetophenone**:$^{1}$H NMR (300 MHz, $CDCl_3$) δ7.99 – 7.92 (m, 2H), 7.66 – 7.59 (m, 1H), 7.50 – 7.41 (m, 2H), 2.57 (s, 3H); $^{13}$C NMR (75 MHz, $CDCl_3$) δ 198.30, 145.80, 136.92, 133.03, 128.21, 77.43, 77.00, 76.58, 26.44. IR $v_{max}$/$cm^{-1}$ 1677

(C=O). In agreement with Sigma Aldrich database https://www.sigmaaldrich.com/spectra/fnmr/FNMR011161.PDF.

**methyl 3-(hydroxymethyl)benzoate (3.8):** Colourless oil, flow conversion 85 %; Rf 0.65 (3:1 EtOAc/Hexanes); $^{1}H$ NMR (300 MHz, $CDCl_3$) δ7.99 (s, 1H), 7.91 (d, *J* = 7.7 Hz, 1H), 7.61 (d, *J* = 7.5 Hz, 1H), 7.49 (t, *J* = 7.6 Hz, 1H), 4.80 (s, 2H), 2.64 (s, 3H).; $^{13}C$ NMR (75 MHz, $CDCl_3$) δ198.15, 141.38, 137.39, 131.57, 128.85, 127.62, 126.63, 77.45, 77.02, 76.60, 64.82, 26.72; IR $v_{max}/cm^{-1}$ 3392 (C-OH), 1675 (C=O)

## References

1. Chung, S.K., *Mechanism of sodium dithionite reduction of aldehydes and ketones.* The Journal of Organic Chemistry, 1981. **46**(26): p. 5457-5458.
2. Minato, H., et al., *Reduction of pyridinium and carbonyl compounds by refluxing sodium dithionite solutions.* Chemistry Letters, 1977. **6**(9): p. 1091-1094.
3. De Vries, J.G. and R.M. Kellogg, *Reduction of aldehydes and ketones by sodium dithionite.* The Journal of Organic Chemistry, 1980. **45**(21): p. 4126-4129.

$^1$H NMR and $^{13}$C NMR spectra of reduced aldehydes:

Phenylmethanol

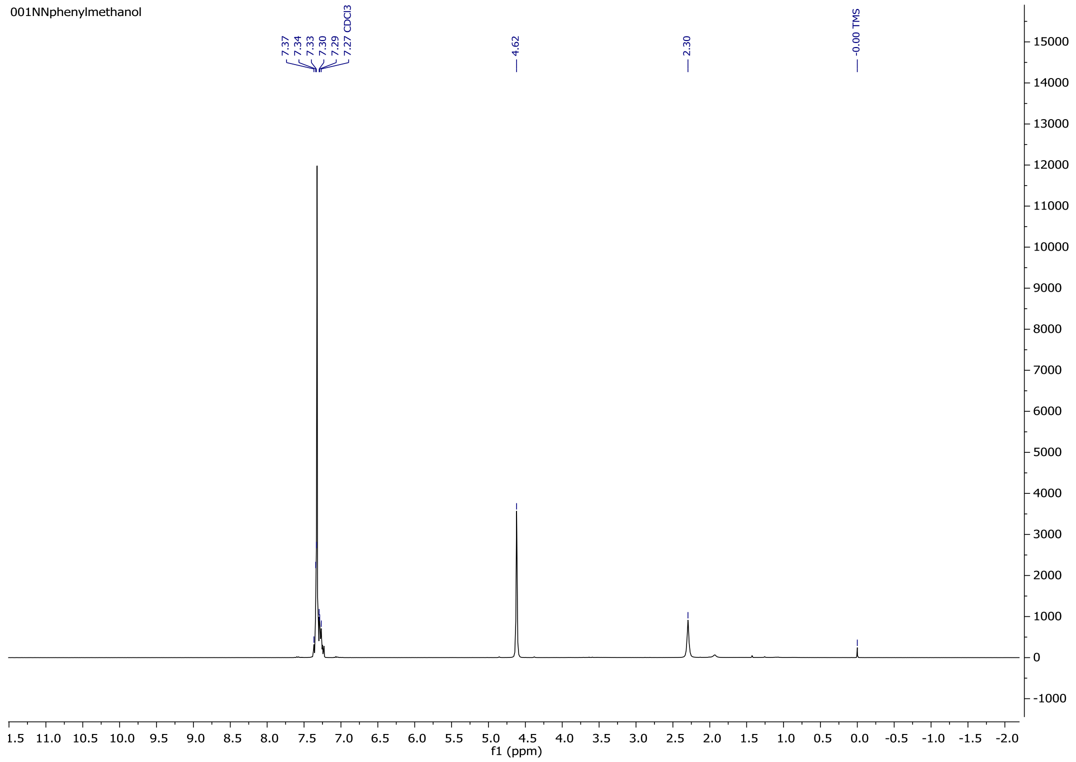


*Figure S1: $^1$H NMR spectrum of phenylmethanol.*

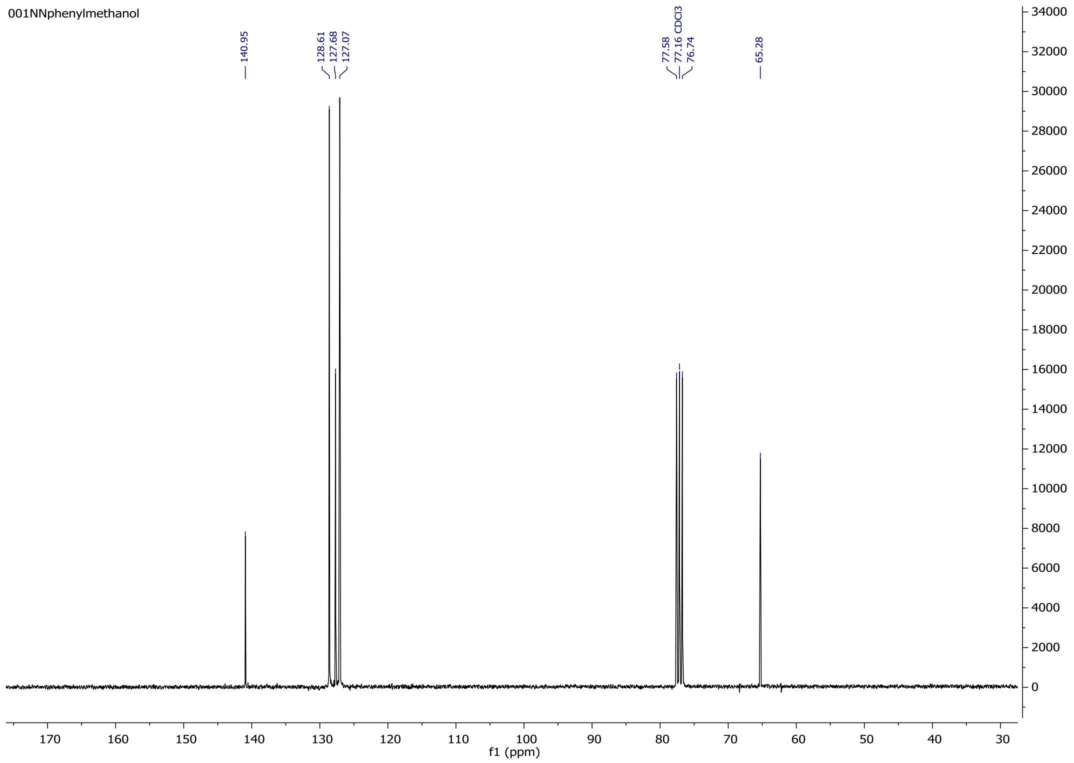


*Figure S2: $^{13}$C NMR spectrum of phenylmethanol.*

## (4-Methylphenyl)methanol

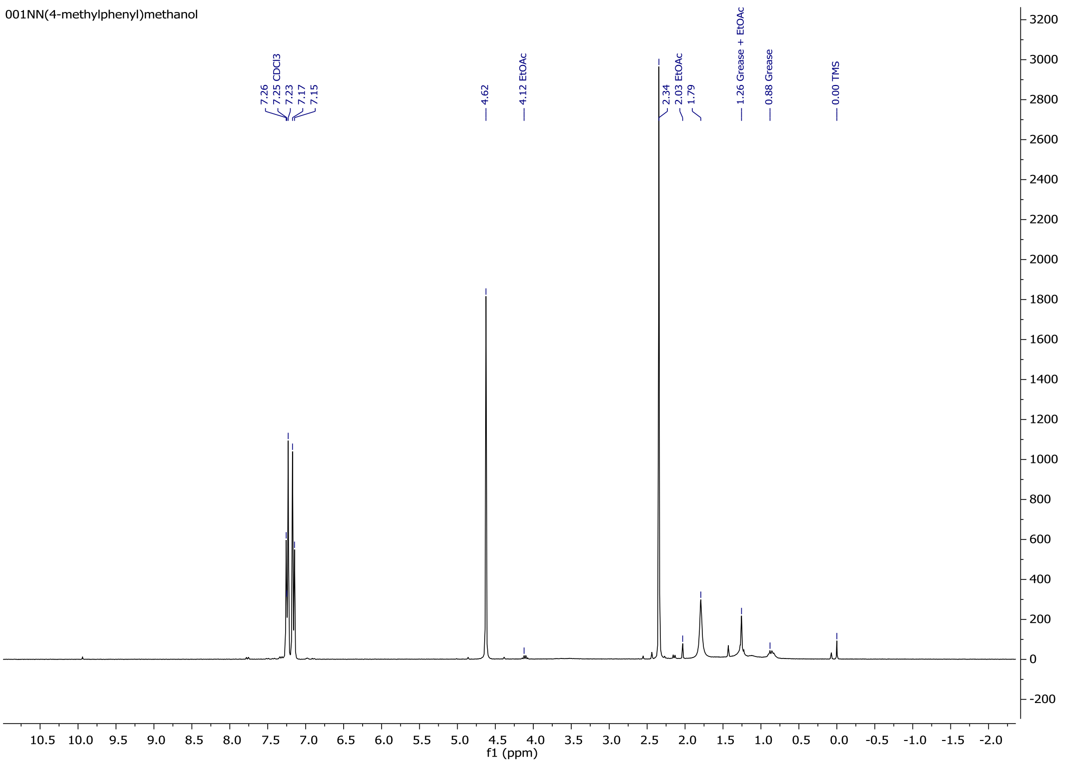


*Figure S3: ¹H NMR spectrum of (4-methylphenyl)methanol.*

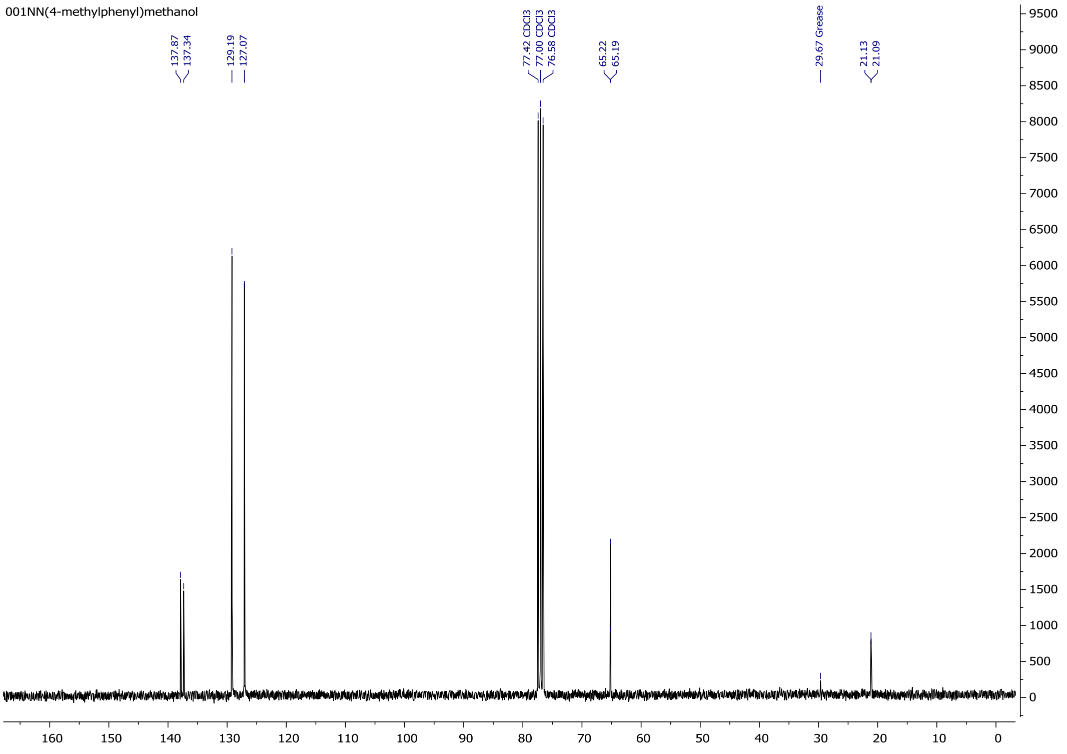


*Figure S4: ¹³C NMR spectrum of (4-methylphenyl)methanol.*

# (4-Methoxyphenyl)methanol

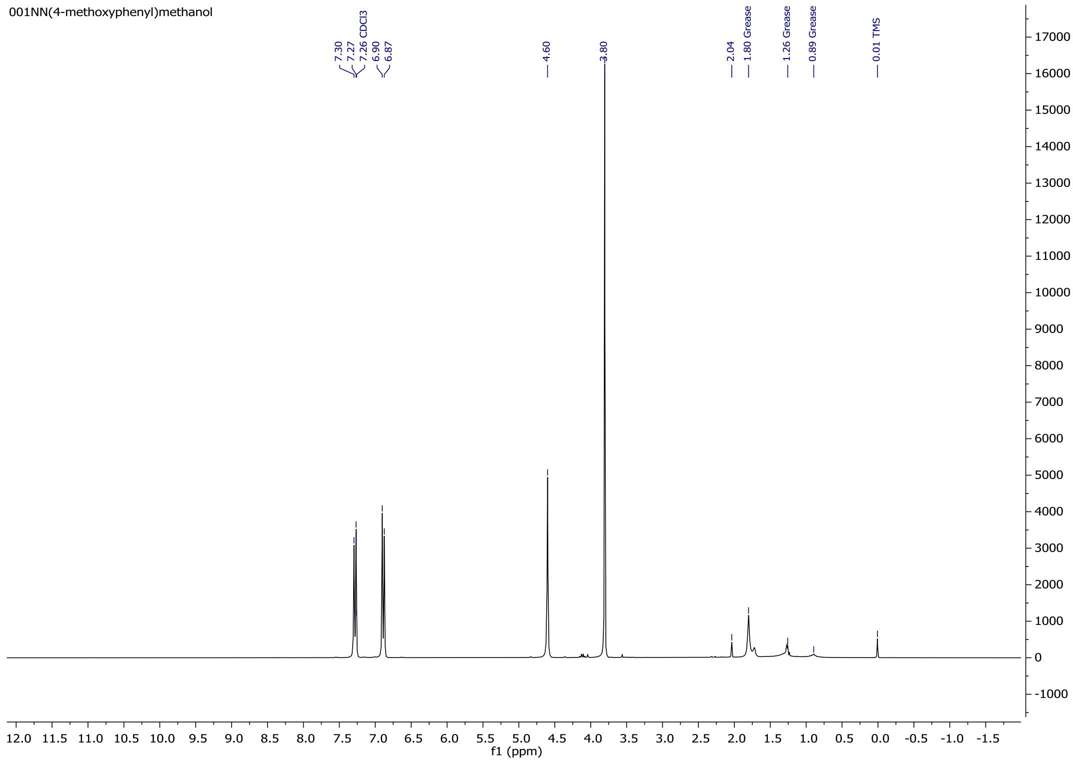


*Figure S5: $^{1}$H NMR spectrum of (4-methoxyphenyl)methanol.*

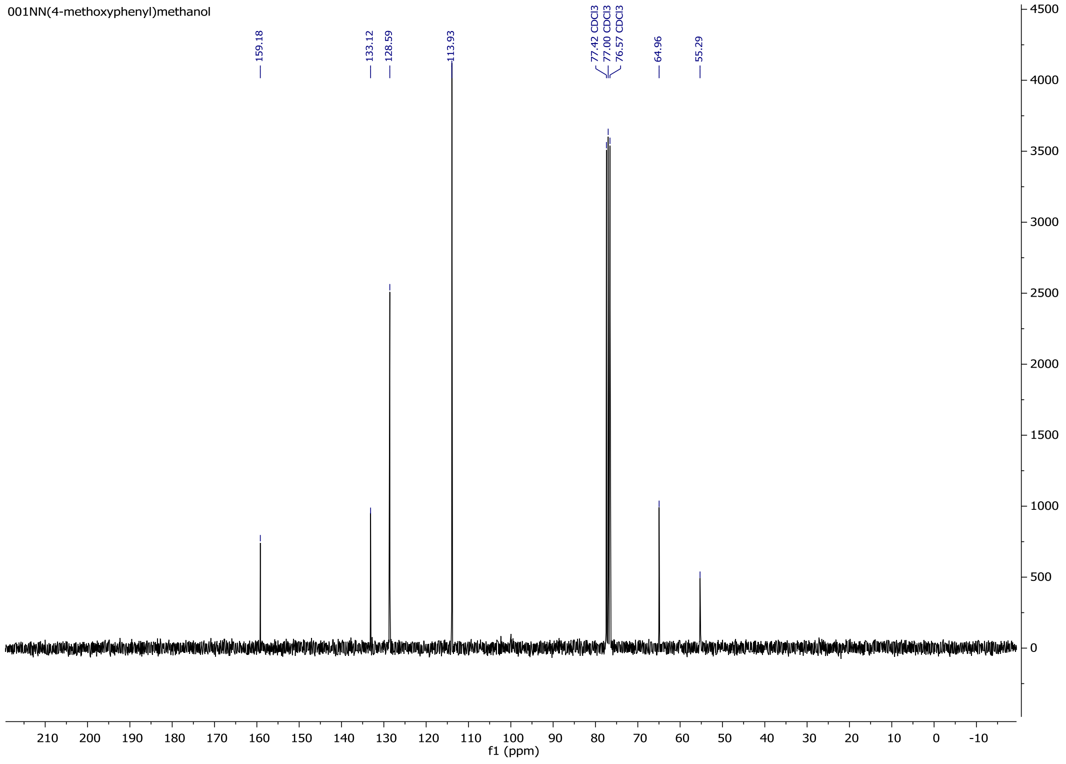


*Figure S4: $^{13}$C NMR spectrum of (4-methoxyphenyl)methanol.*

# (3-Methoxyphenyl)methanol

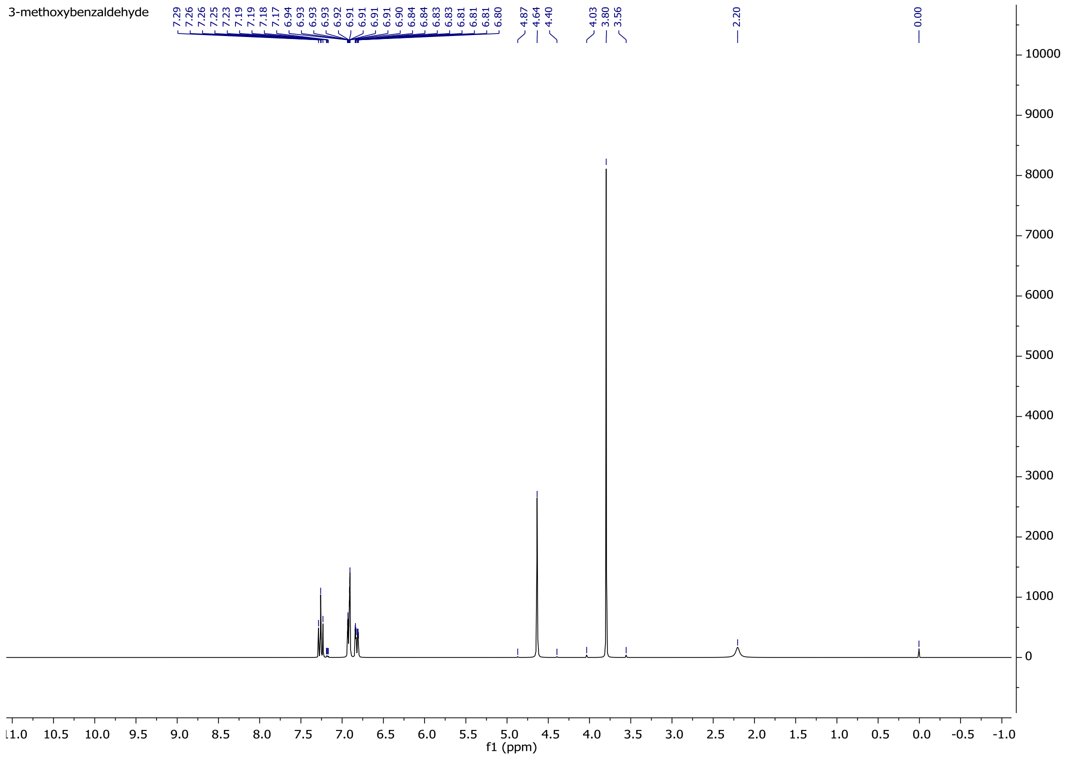


*Figure S5: $^{1}$H NMR spectrum of (3-methoxyphenyl)methanol.*

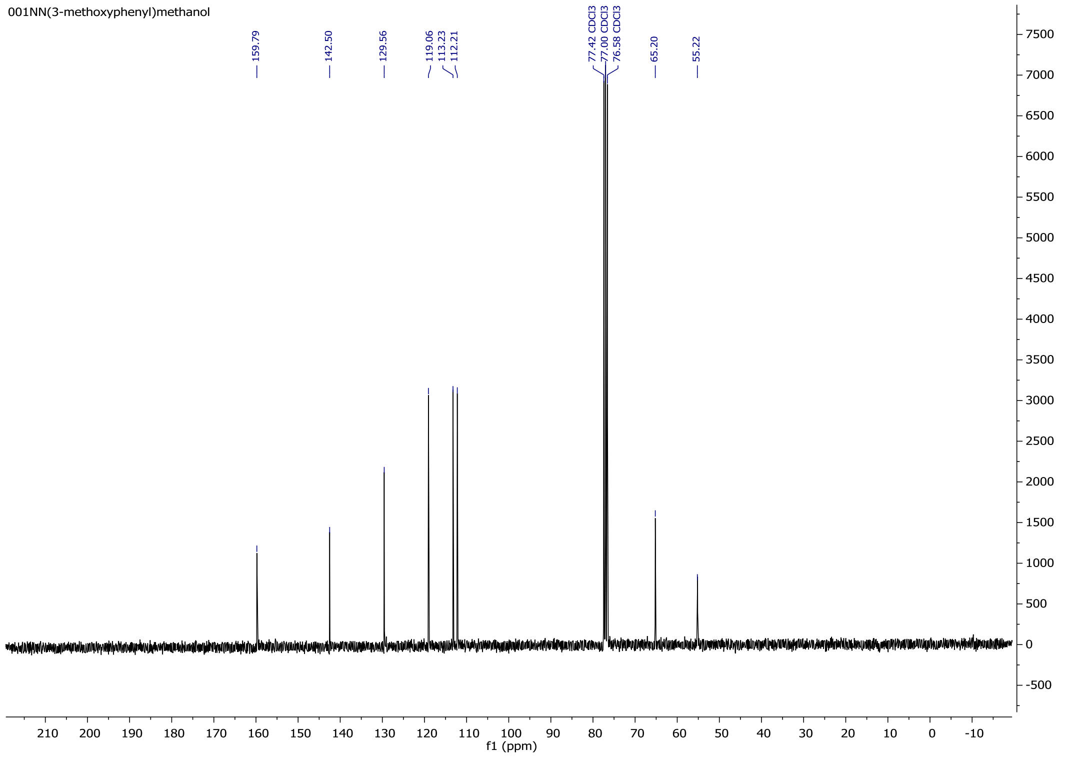


*Figure S6: $^{13}$C NMR spectrum of (3-methoxyphenyl)methanol.*

# (4-Chlorophenyl)methanol

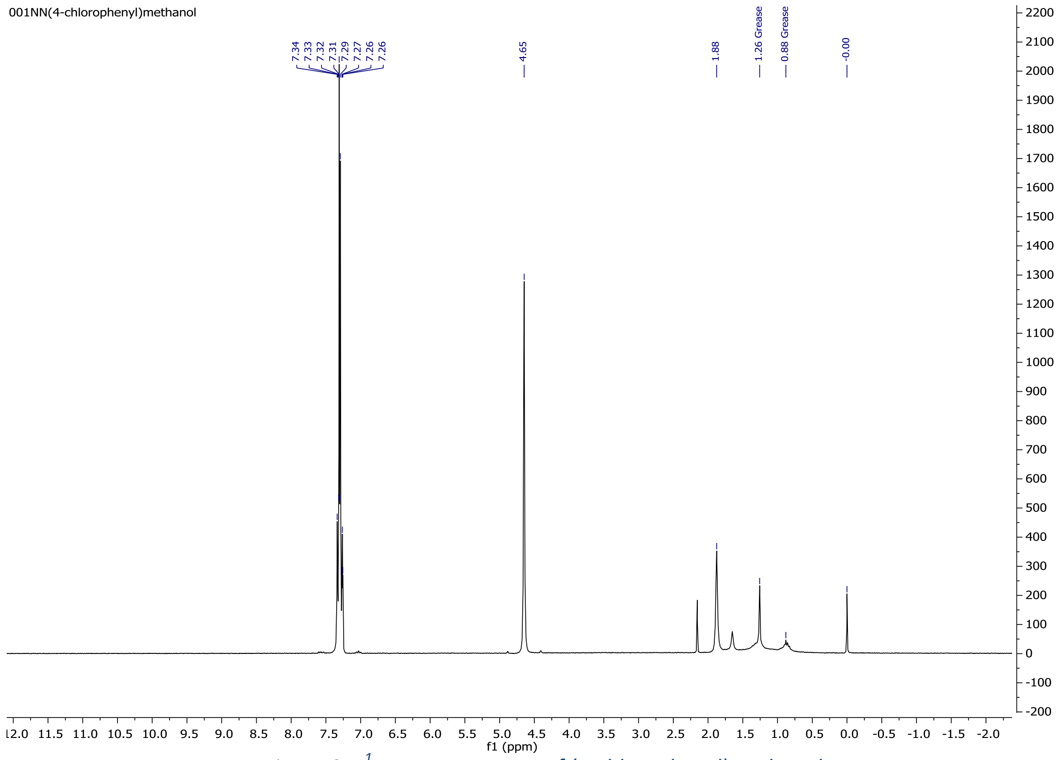


*Figure S7: $^{1}$H NMR spectrum of (4-chlorophenyl)methanol.*

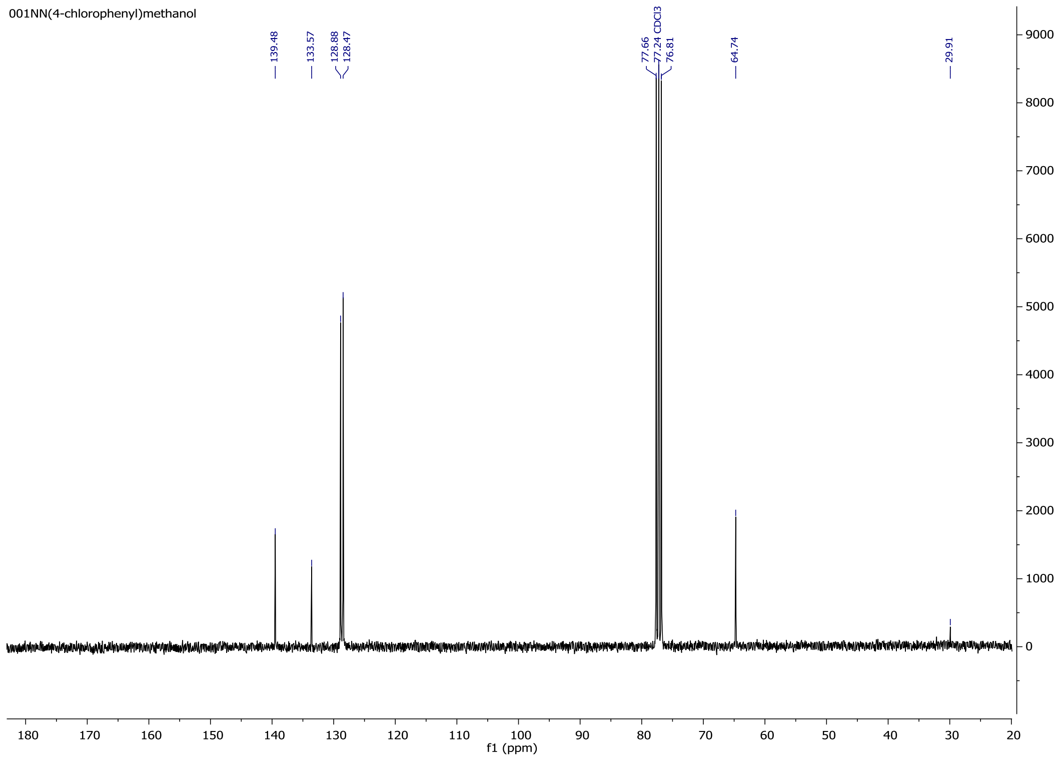


*Figure S8: $^{13}$C NMR spectrum of (4-chlorophenyl)methanol.*

## (3-Bromophenyl)methanol

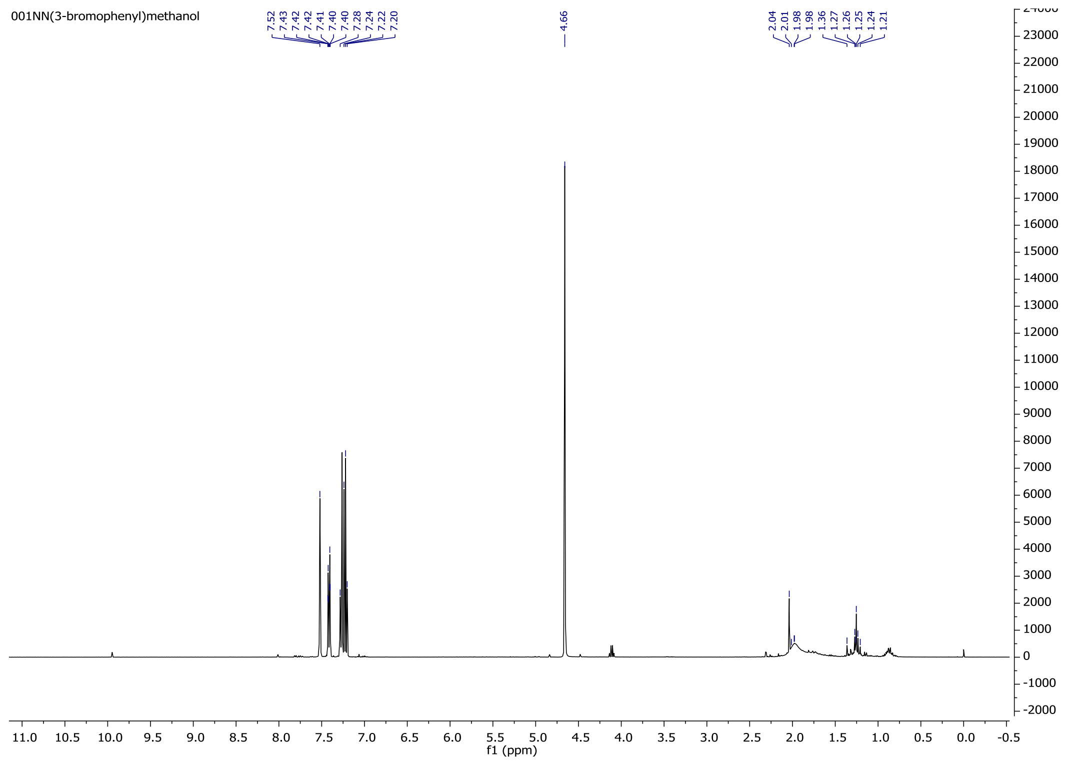


*Figure S9: ¹H NMR spectrum of (3-bromophenyl)methanol.*

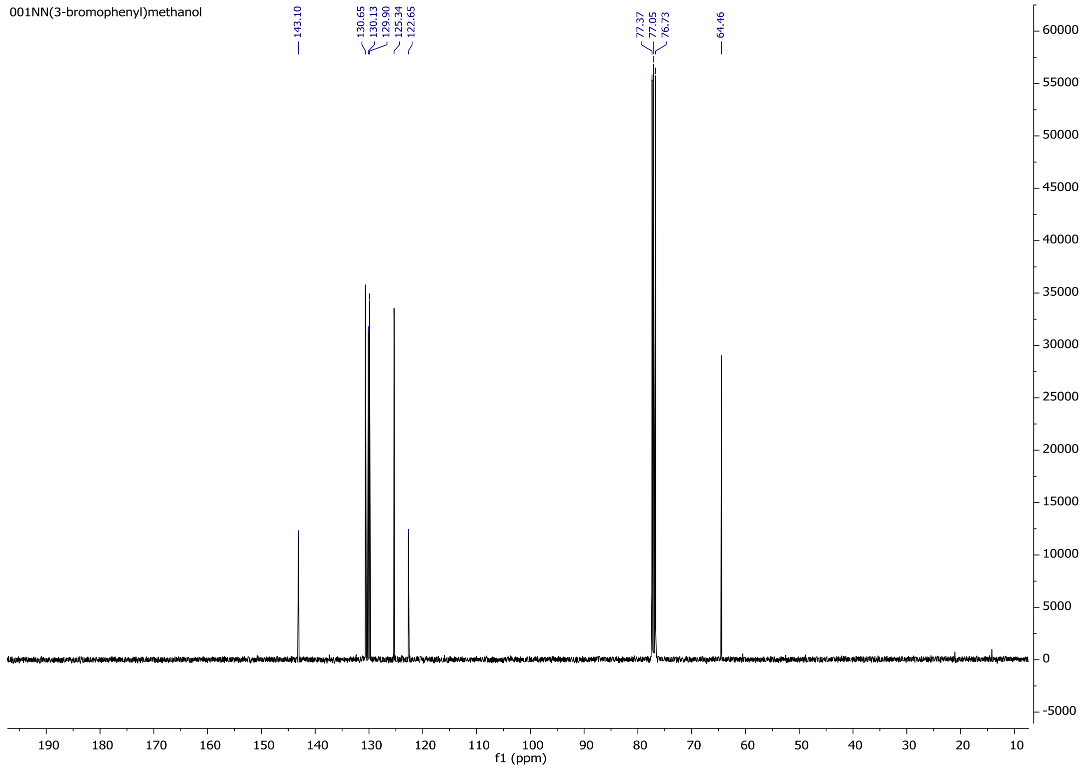


*Figure S10: ¹³C NMR spectrum of (3-bromophenyl)methanol.*

(2-Bromophenyl)methanol

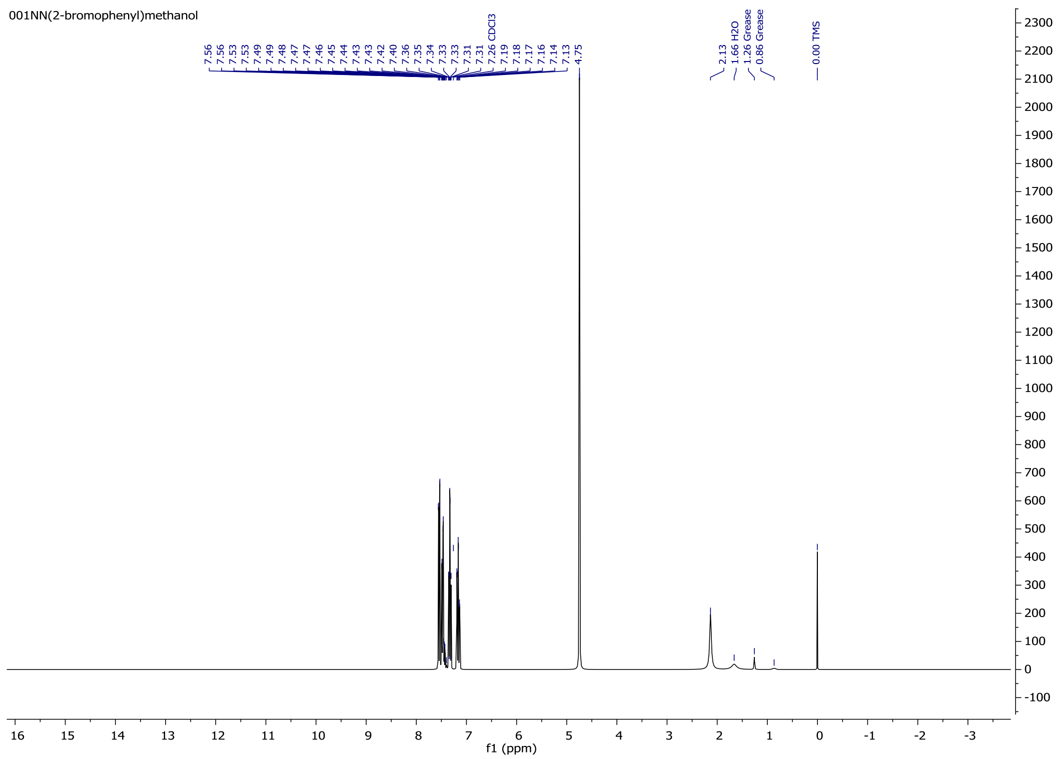


*Figure S12: $^{1}$H NMR spectrum of (2-bromophenyl)methanol.*

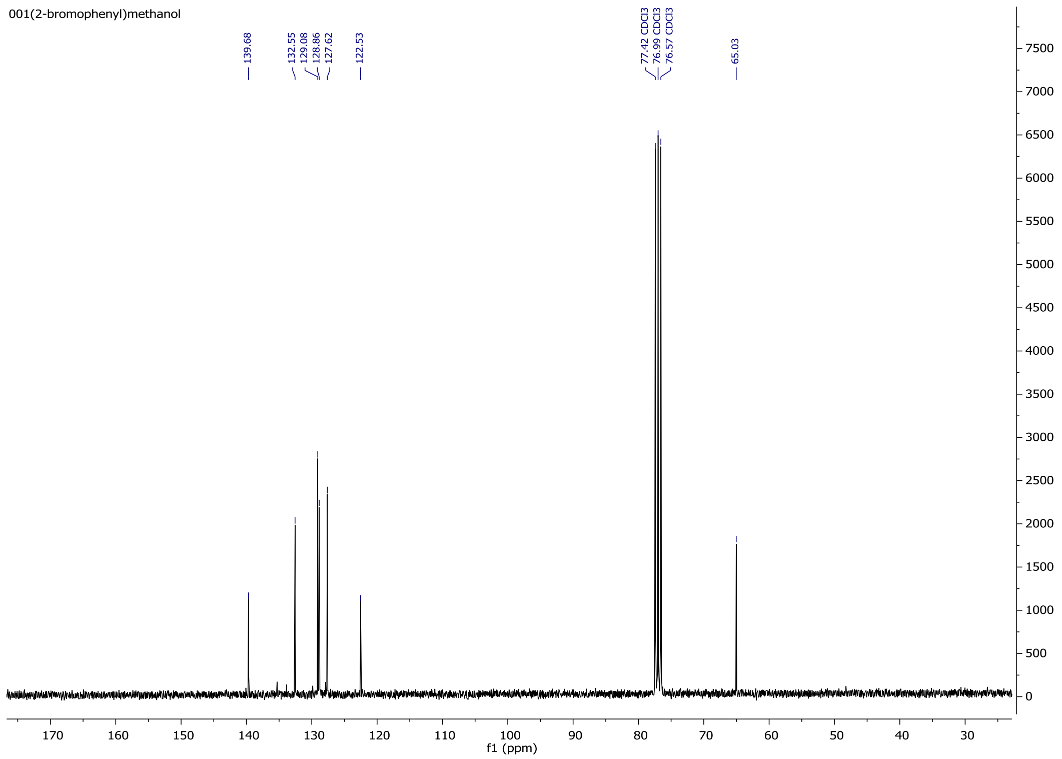


*Figure S11: $^{13}$C NMR spectrum of (2-bromophenyl)methanol.*

# Pyridin-3-ylmethanol

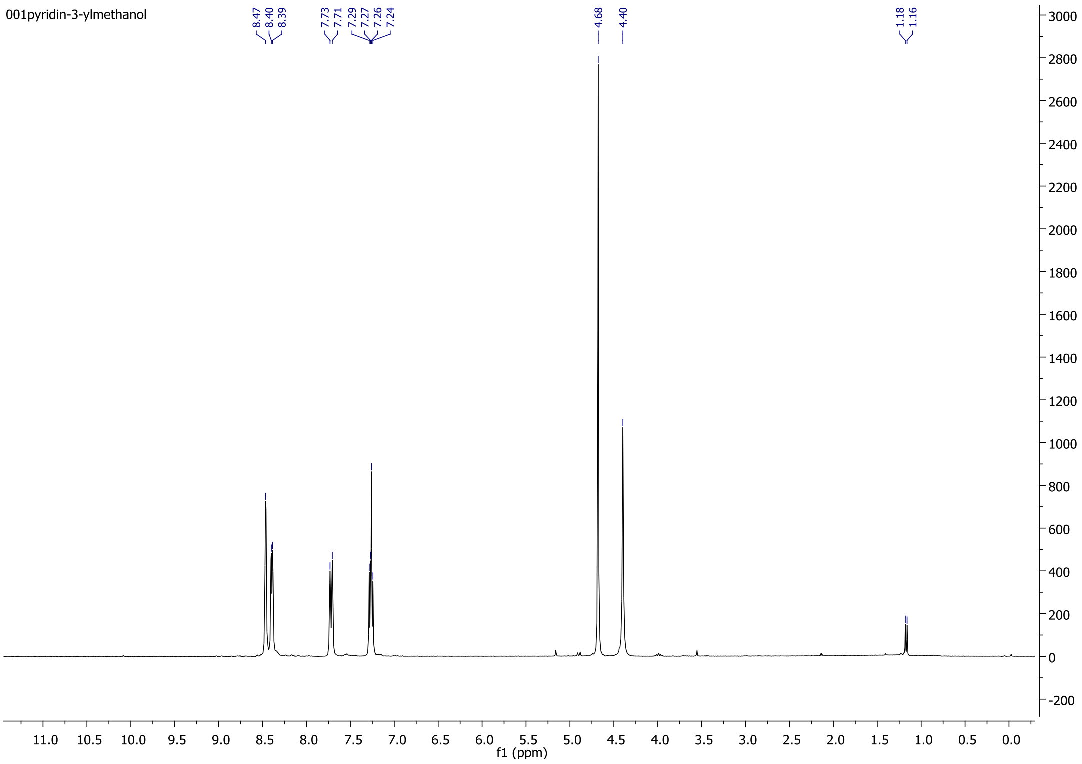


*Figure S13: $^{1}$H NMR spectrum of pyridin-3-ylmethanol.*

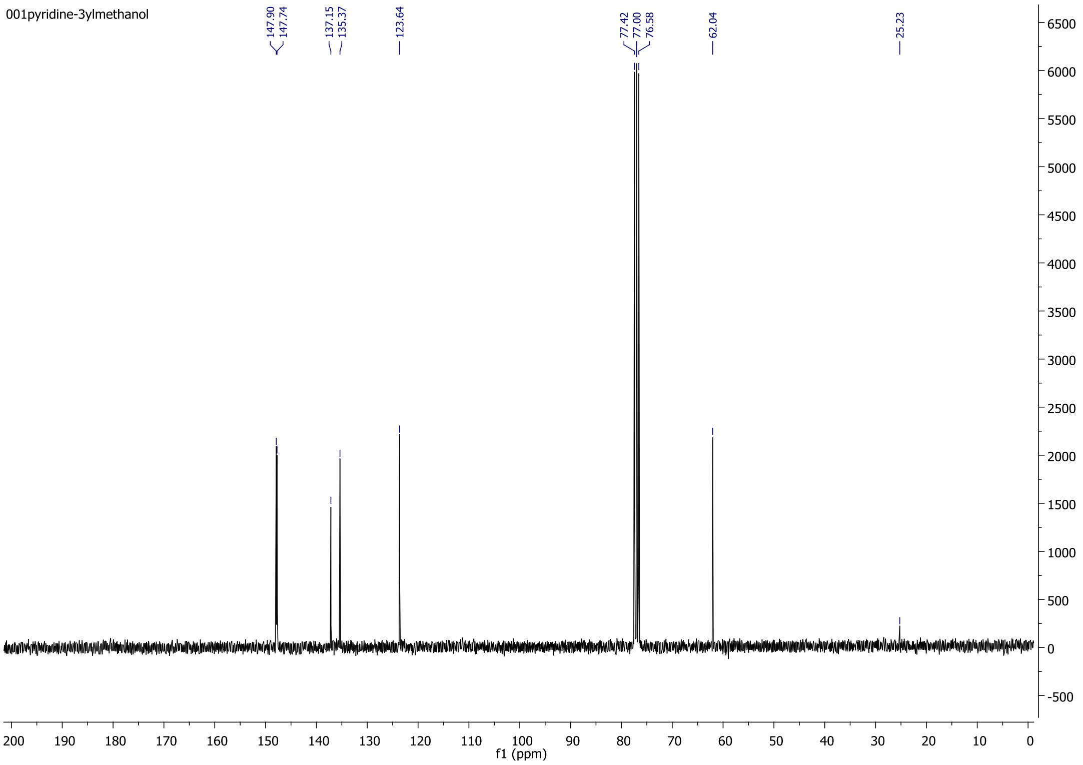


*Figure S14: $^{13}$C NMR spectrum of pyridine-3-ylmethanol.*

## Heptanol

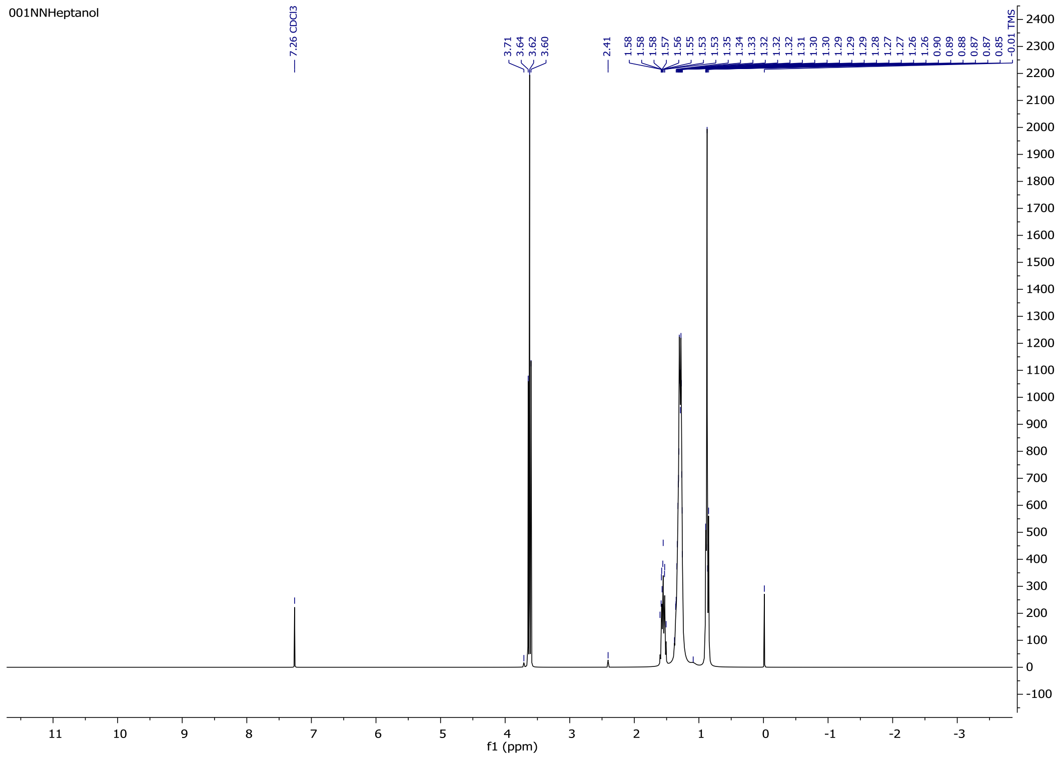


*Figure S16: $^{1}$H NMR spectrum of heptanol.*

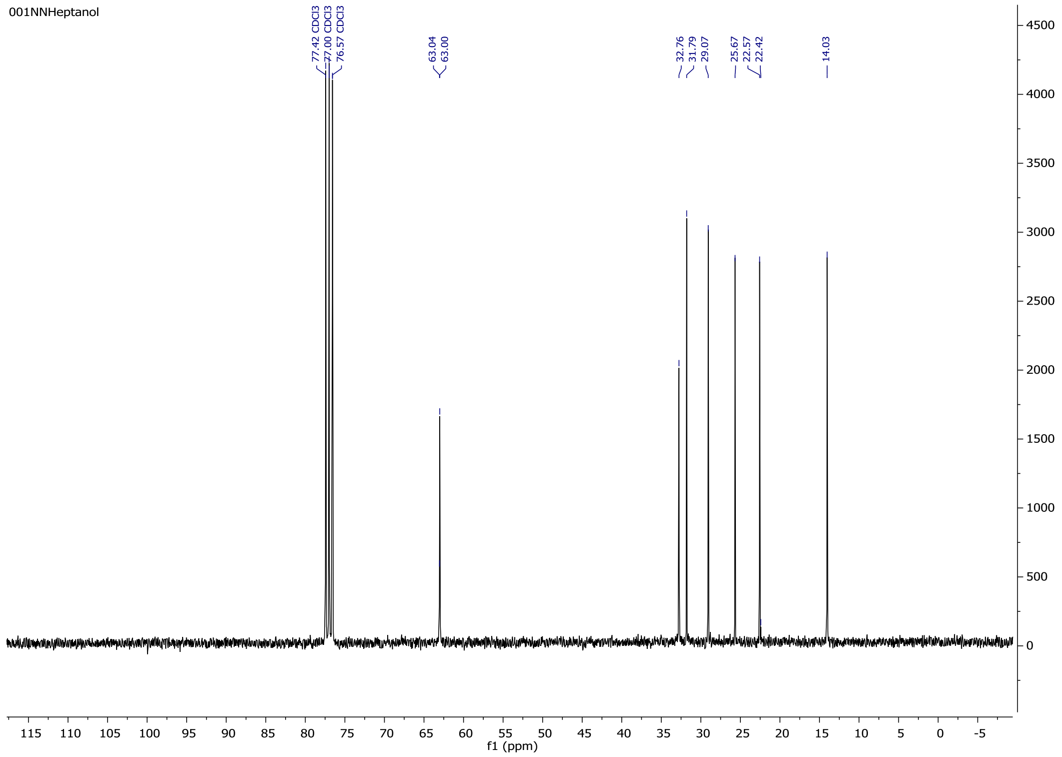


*Figure S15: $^{13}$C NMR spectrum of heptanol.*

# Hexanol

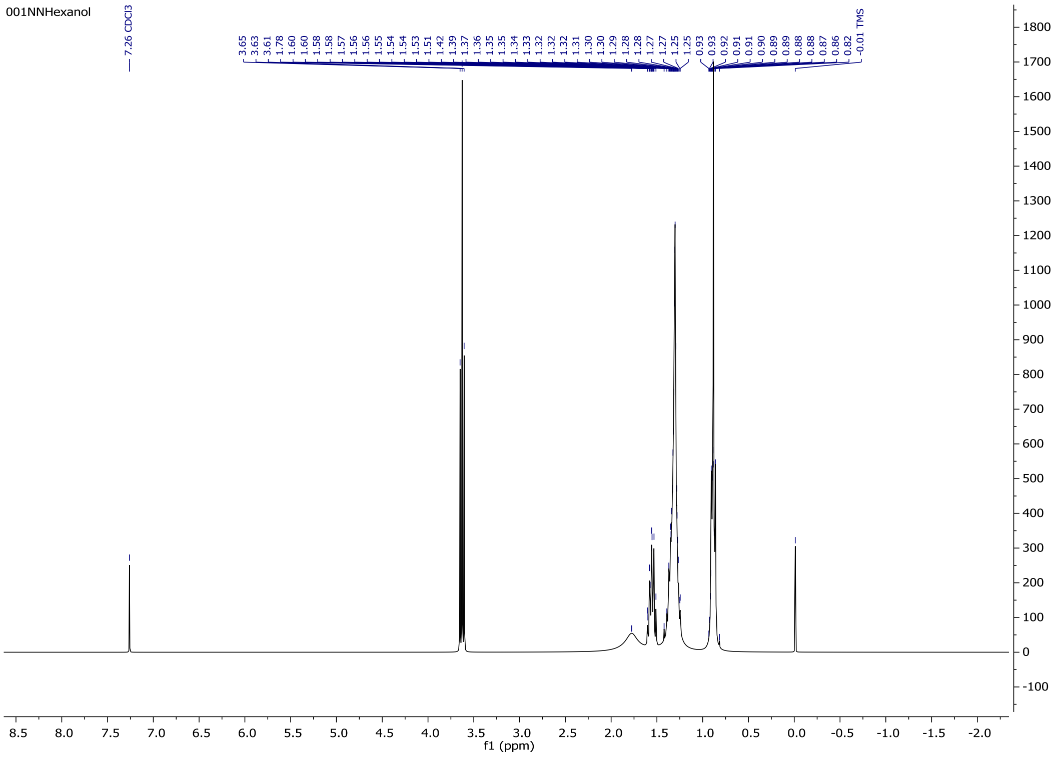


*Figure S18: $^1$H NMR spectrum of hexanol.*

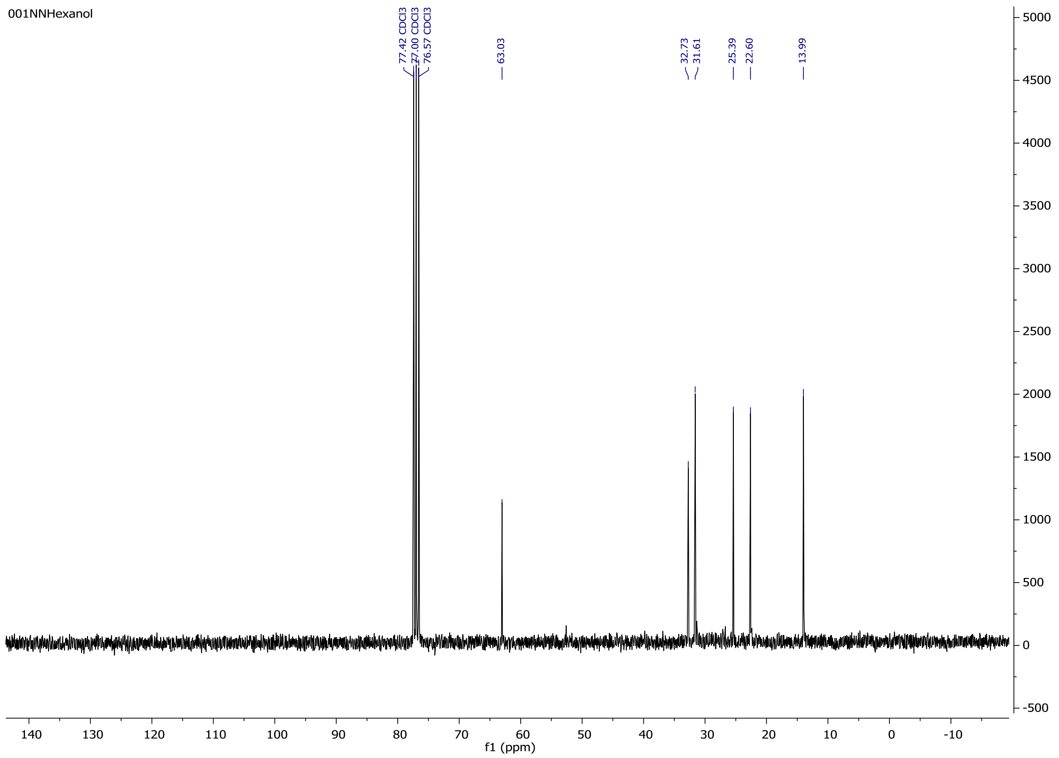


*Figure S17: $^{13}$C NMR spectrum of heptanol.*

$^1H$ and $^{13}C$ NMR for reduced ketones in batch:

# 1-(4-Chlorophenyl)ethanol and 4-chloroacetophenone mixture

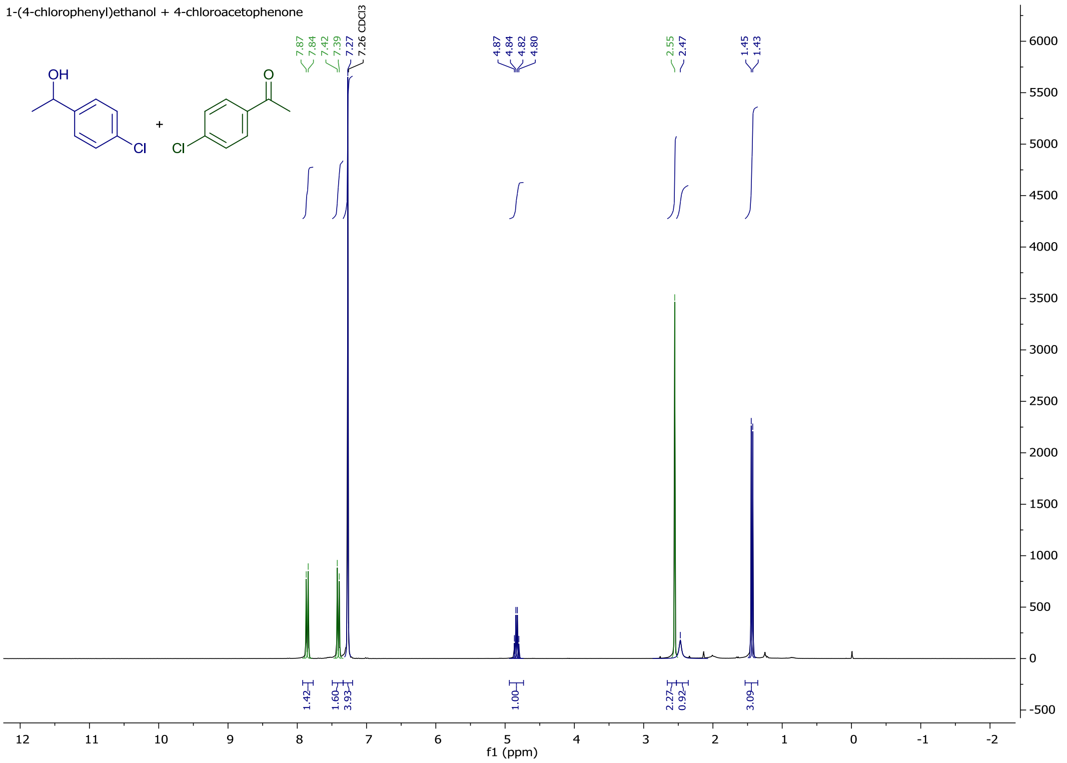


*Figure S19: $^1H$ NMR spectrum of 1-(4-chlorophenyl)ethanol and 4-chloroacetophenone.*

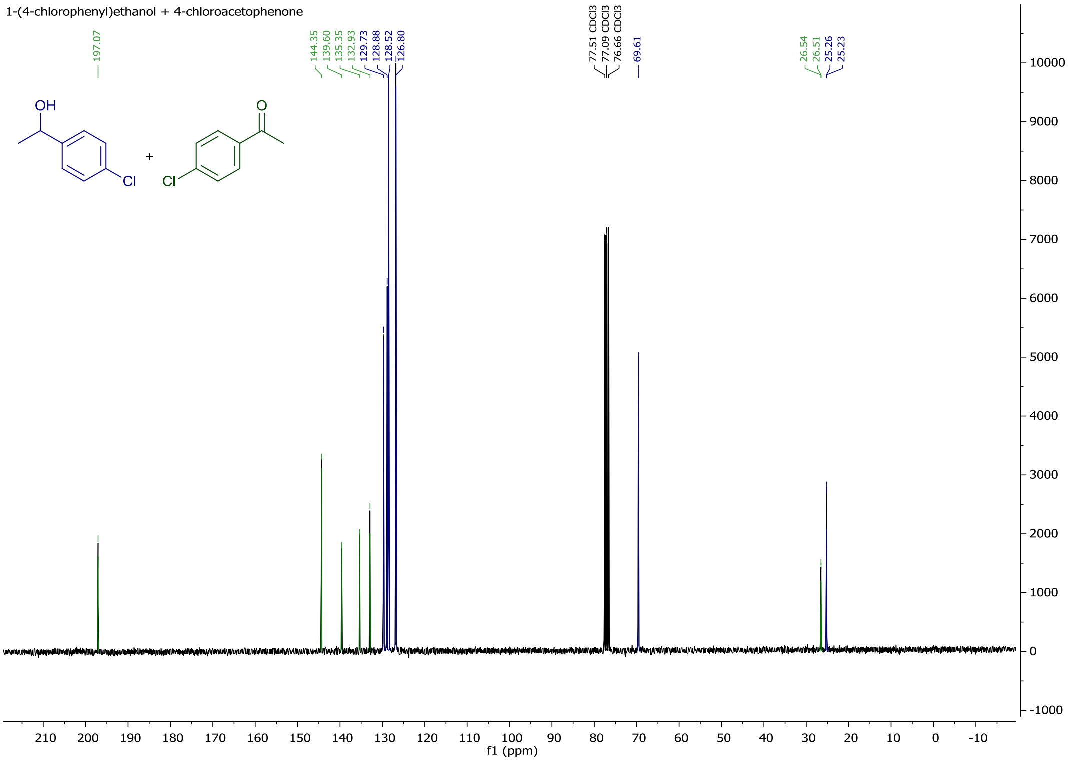


*Figure S20: $^{13}C$ NMR spectrum of 1-(4-chlorophenyl)ethanol and 4-chloroacetophenone.*

# 1-(*p*-Tolylethanol) and 4-methylacetophenone mixture

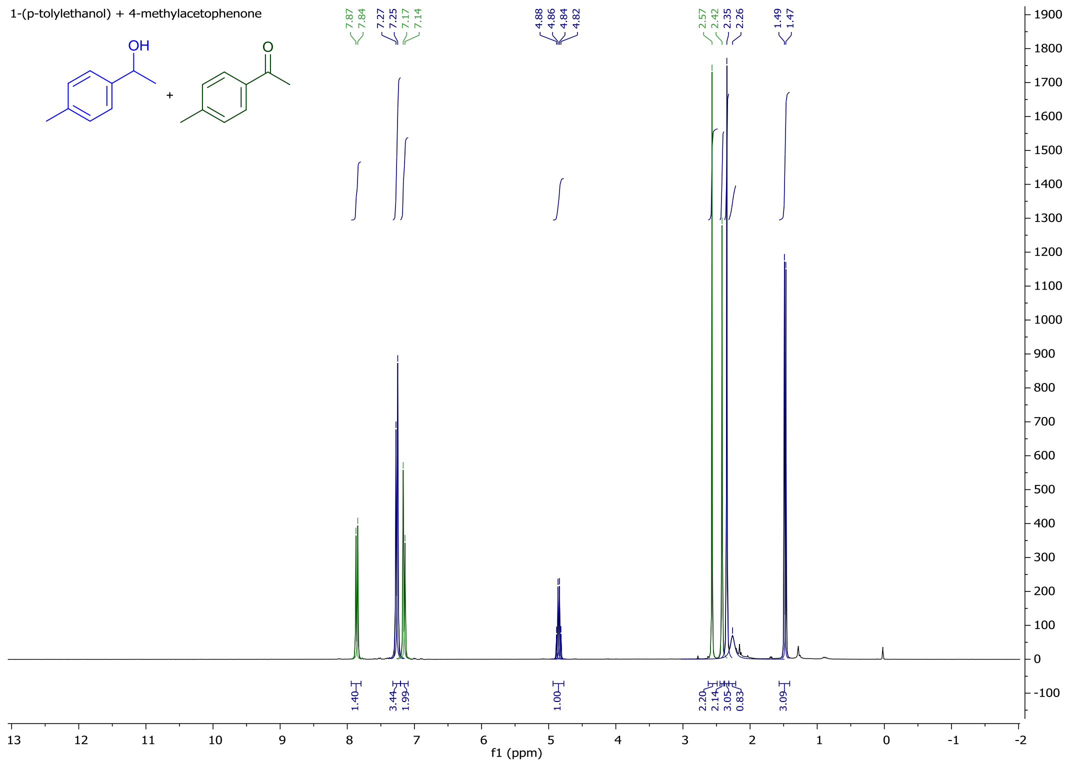


*Figure S22: $^{1}$H NMR spectrum of 1-(4-methylphenyl) ethanol and 4-methylacetophenone.*

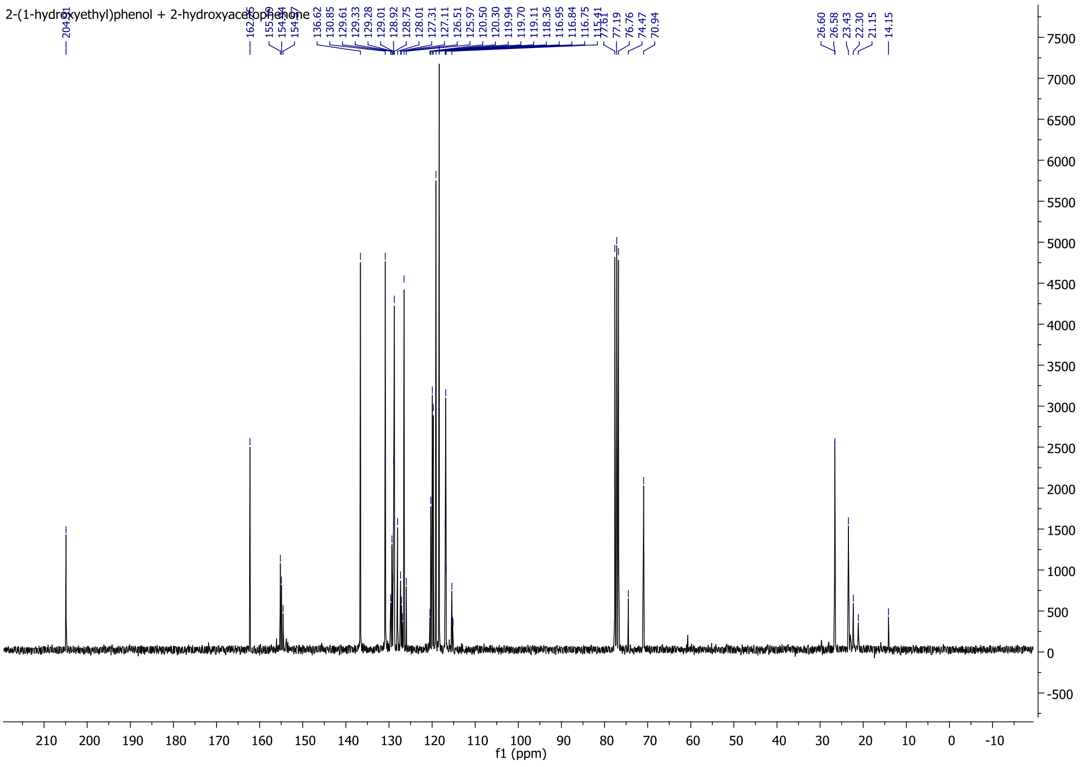


*Figure S21: $^{13}$C NMR spectrum of 1-(4-methylphenyl) ethanol and 4-methylacetophenone*

## 2-(1-Hydroxyethyl)phenol and 2-hydroxyacetophenone mixture

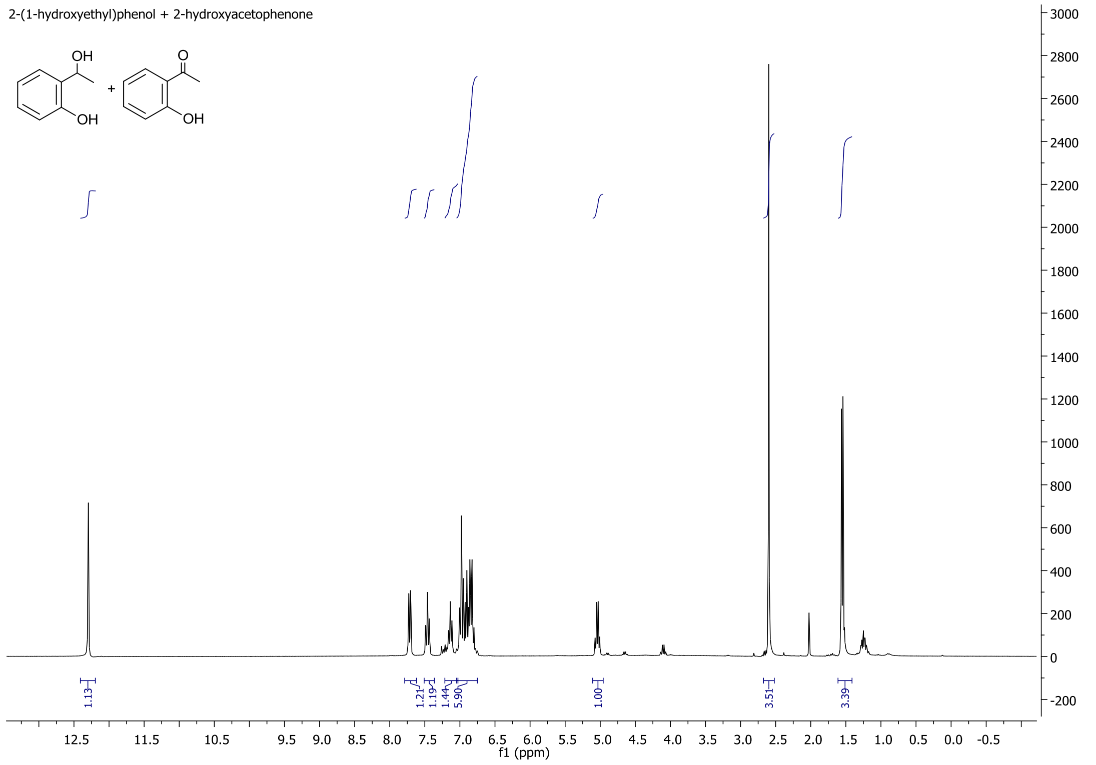


*Figure S23: $^{1}$H NMR spectrum of 1-(2-hydroxyphenyl)ethanol and 2-hydroxyacetophenone.*

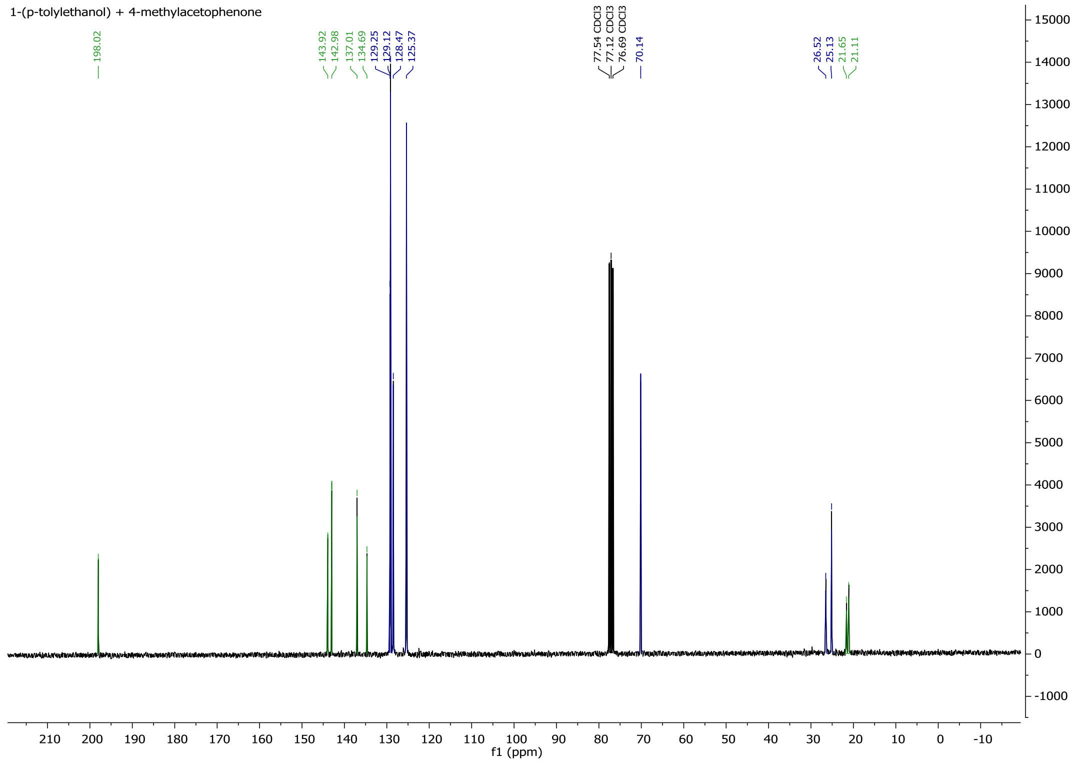


*Figure S24: $^{13}$C NMR spectrum of 1-(2-hydroxyphenyl)ethanol and 2-hydroxyacetophenone.*

# Cyclohexanol

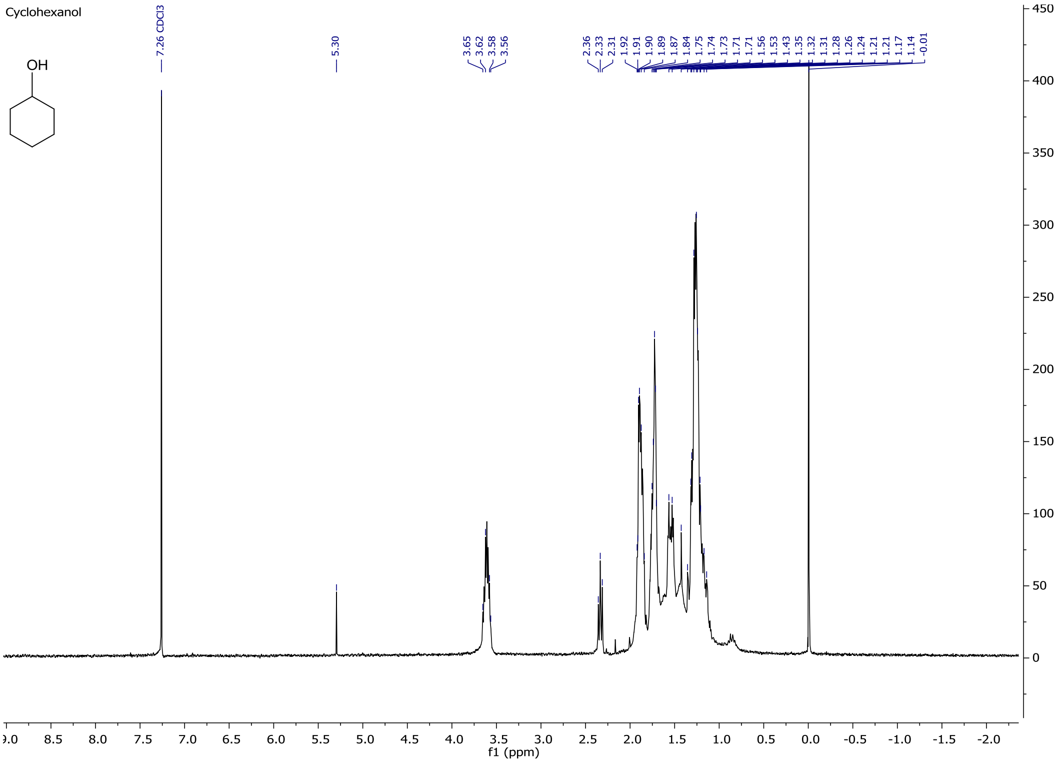


*Figure S26: $^{1}$H NMR spectrum of cyclohexanol.*

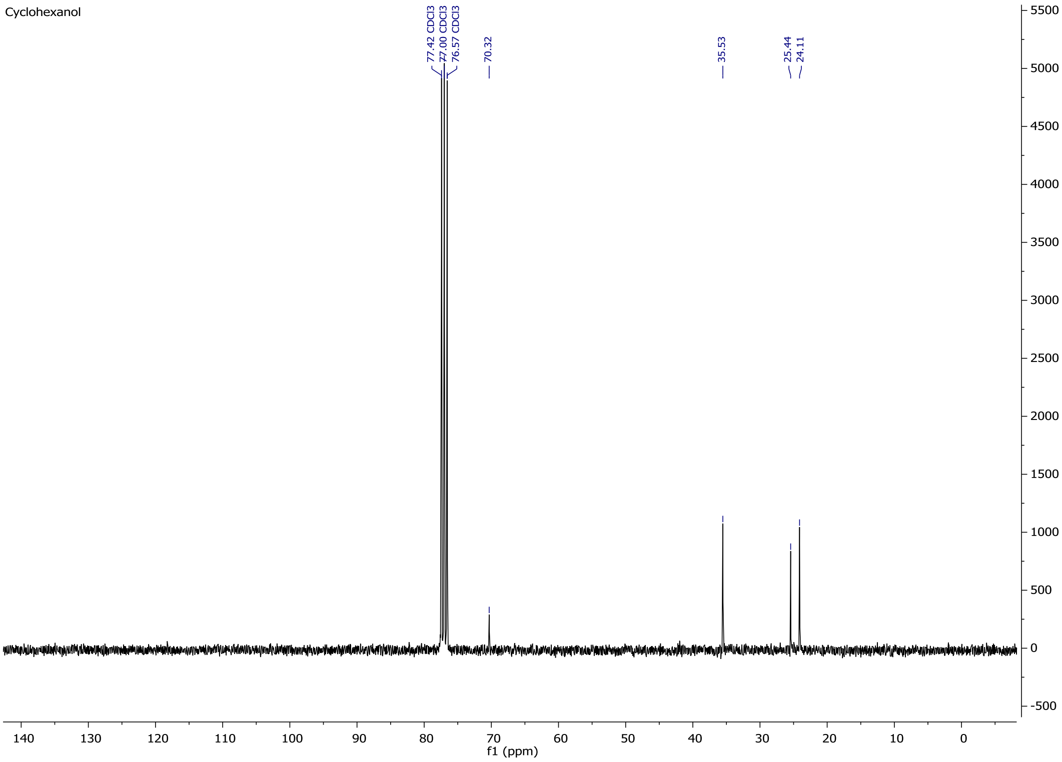


*Figure S25: $^{13}$C NMR spectrum of cyclohexanol.*

## 3-Heptanol and 3-heptanone mixture

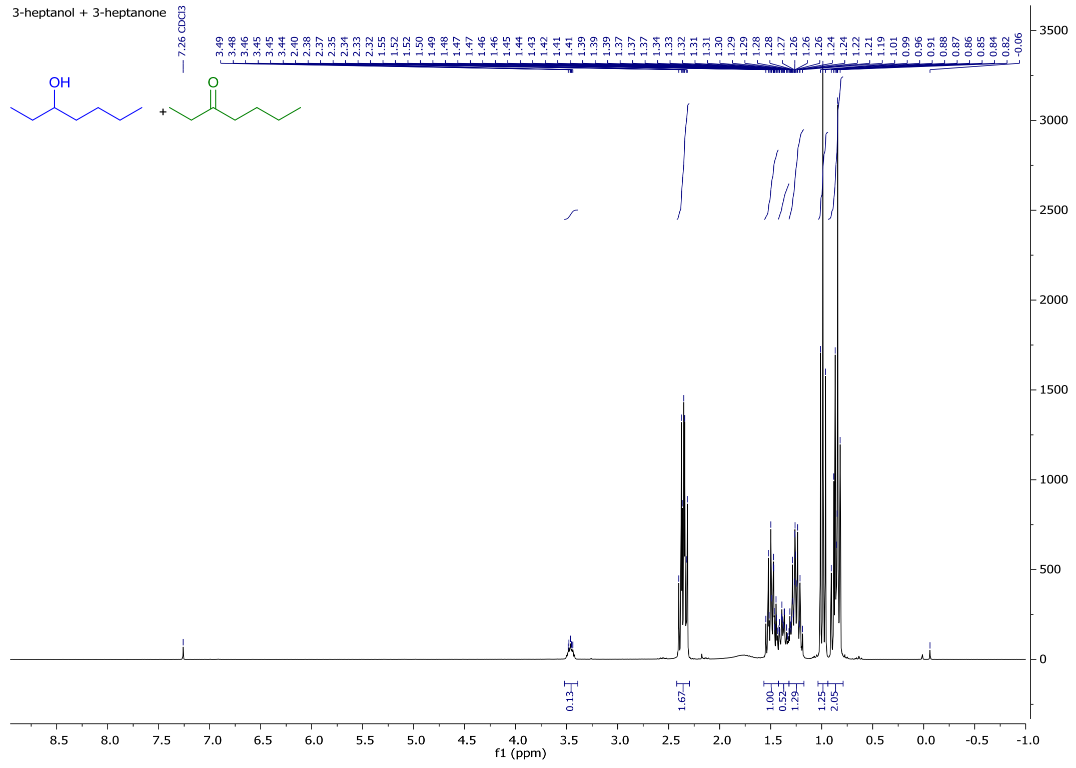


*Figure S27: $^{1}$H NMR spectrum of 3-hetanol and 3-heptanone.*

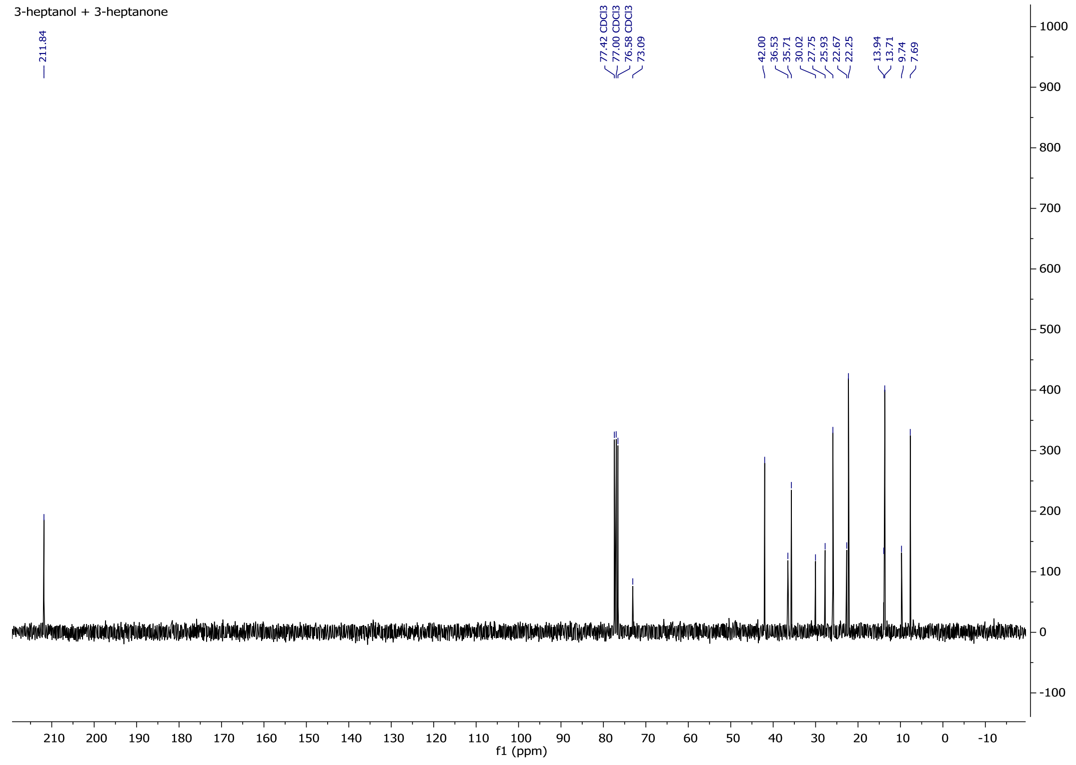


*Figure S30: $^{13}$C NMR spectrum of 3-hetanol and 3-heptanone.*

1 -Phenylethanol and acetophenone mixture

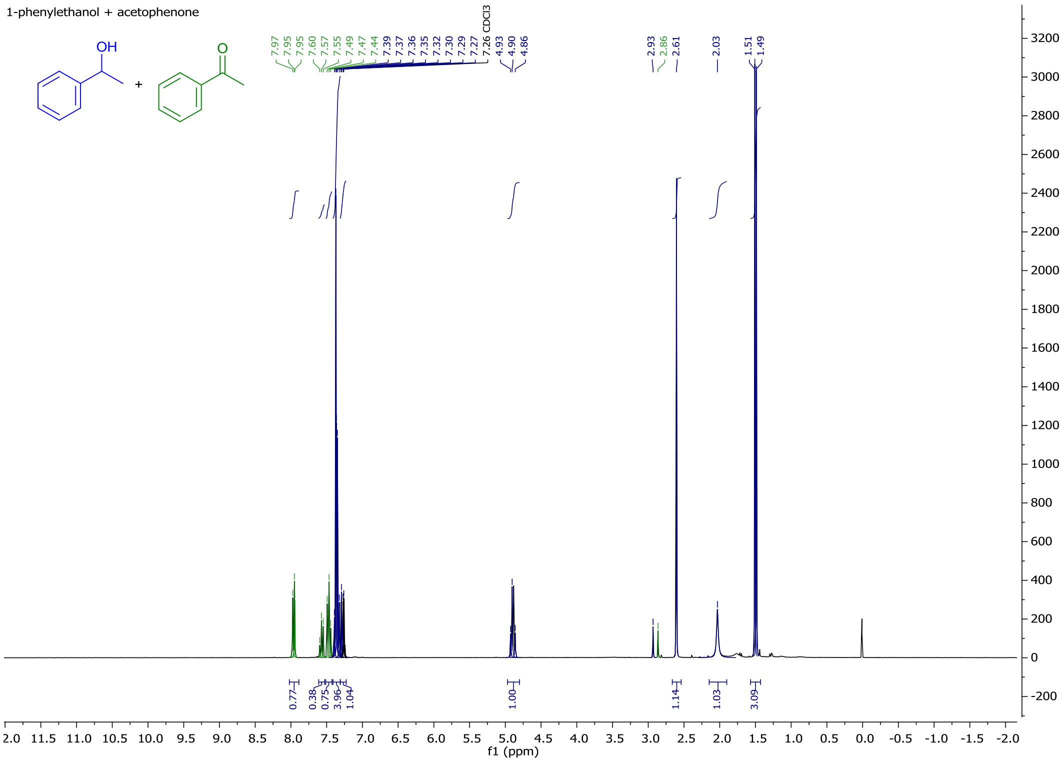


*Figure S28: $^{1}$H NMR spectrum for 1-phenylethanol and acetophenone.*

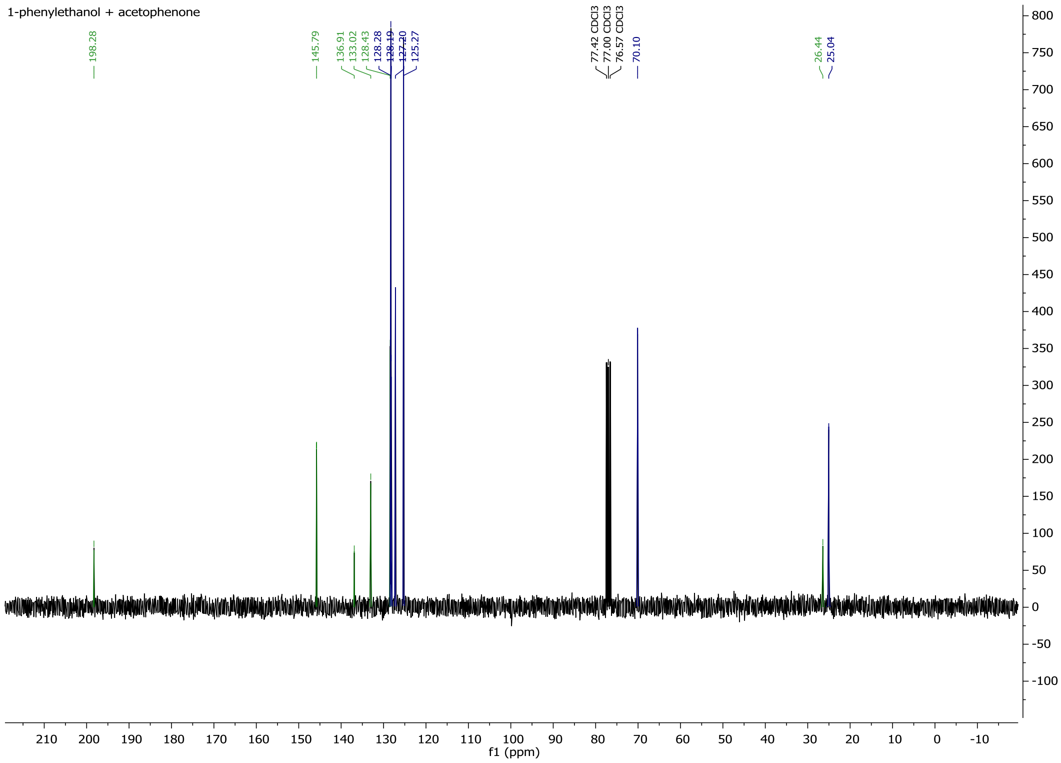


*Figure S29: $^{13}$C NMR spectrum of 1-phenylethanol and acetophenone.*

# $^1$H NMR for reduced ketones in flow:

## 1-(4-Chlorophenyl)ethanol and 4-chloroacetophenone mixture

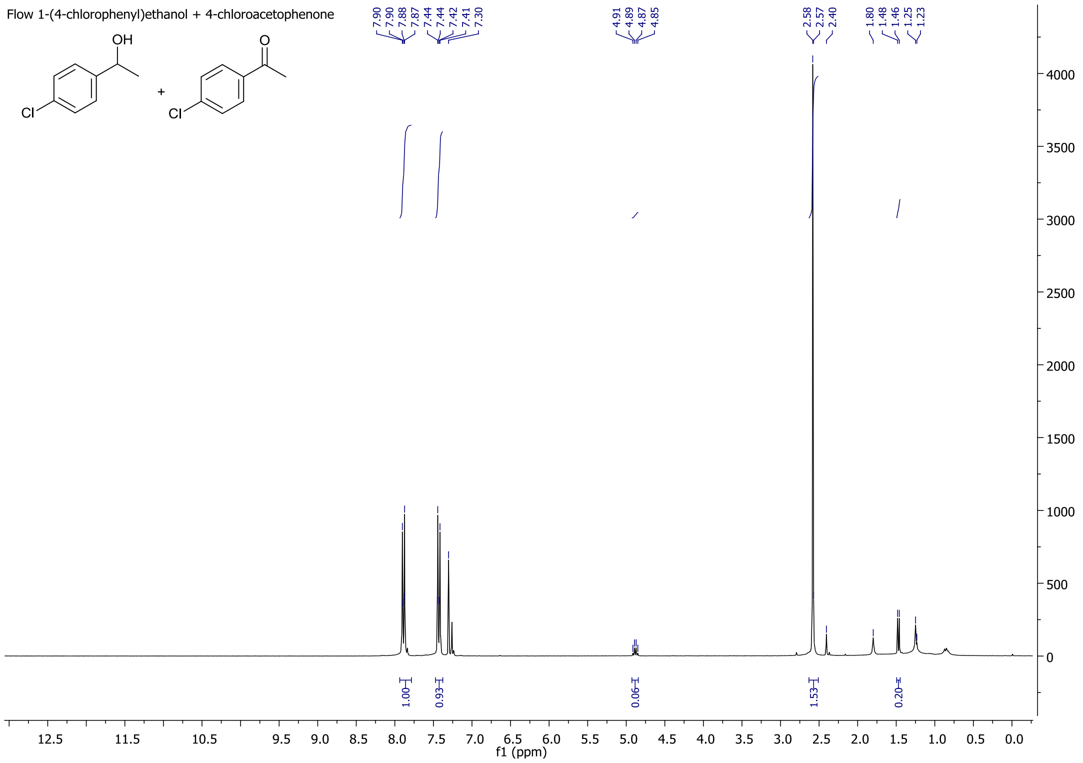


*Figure S30: $^1$H NMR spectrum of 1-phenylacetophenone and acetophenone.*

## 1-(4-Methylphenyl)ethanol and 4-methylacetophenone mixture

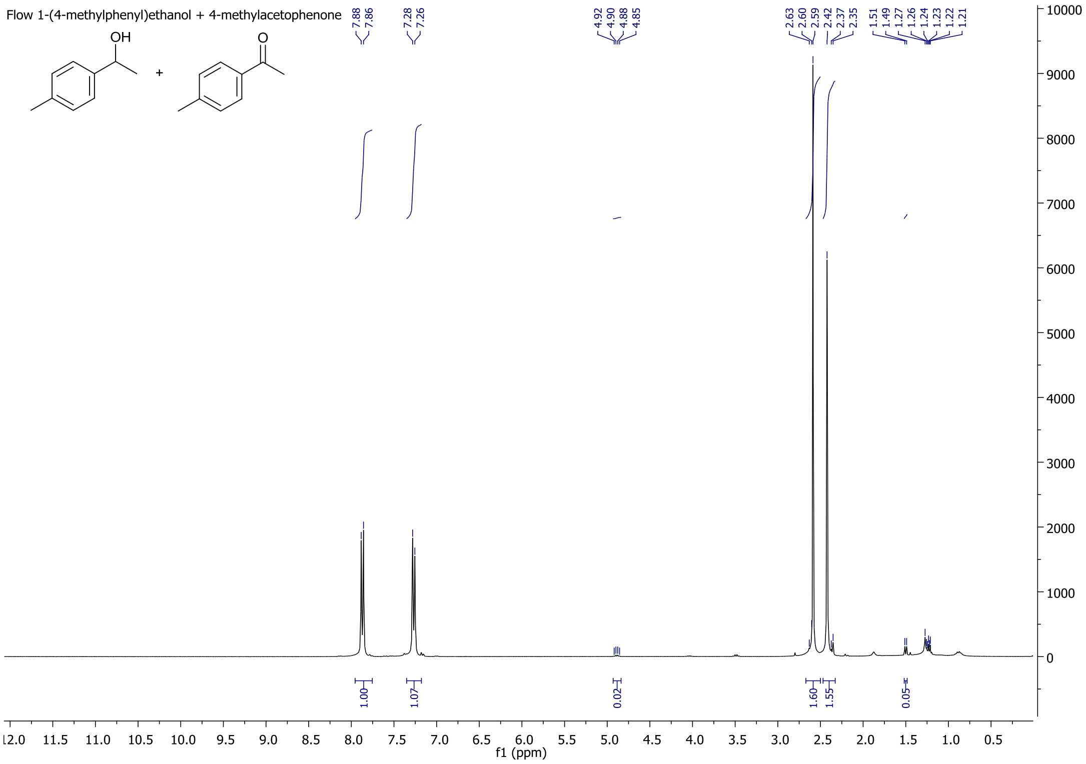


*Figure S31: $^1$H NMR spectrum of 1-(4-methylphenyl)ethanol and 4-methylacetophenone.*

## 1-(2-Hydroxyphenyl)ethanol and 2-hydroxyacetophenone mixture

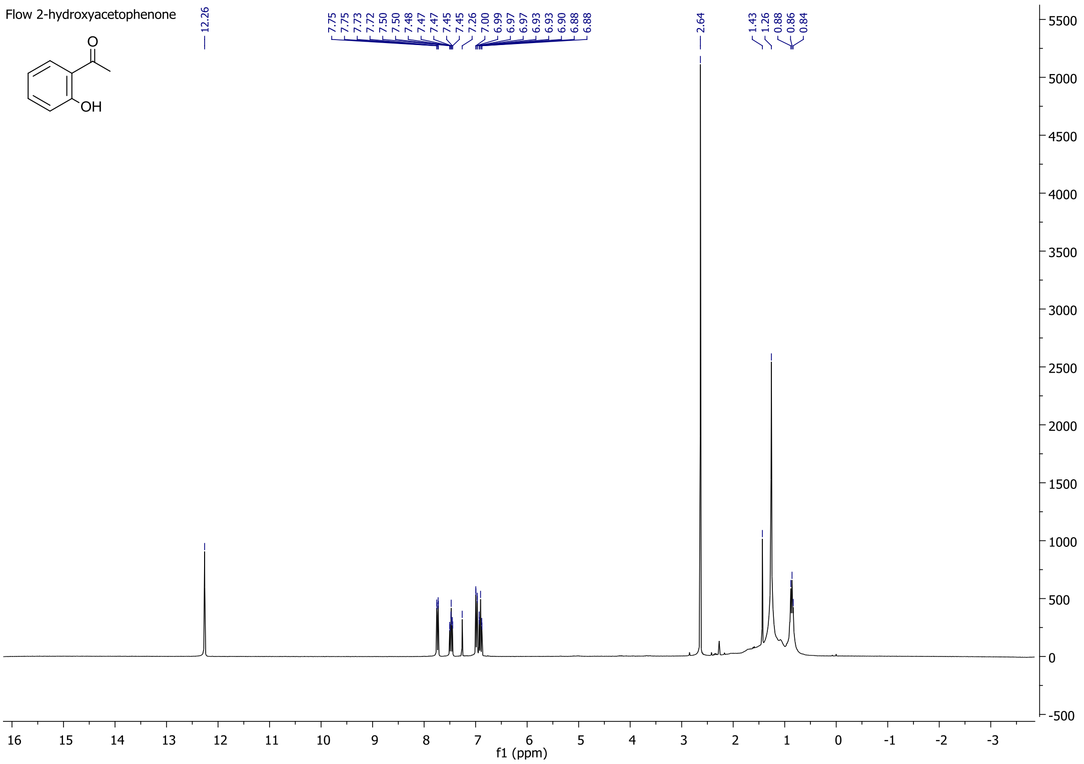


*Figure S32: $^{1}$H NMR spectrum of 1-(2-hydroxyphenyl)ethanol and 2-hydroxyacetophenone mixture.*

## Cyclohexanol and cyclohexanone mixture

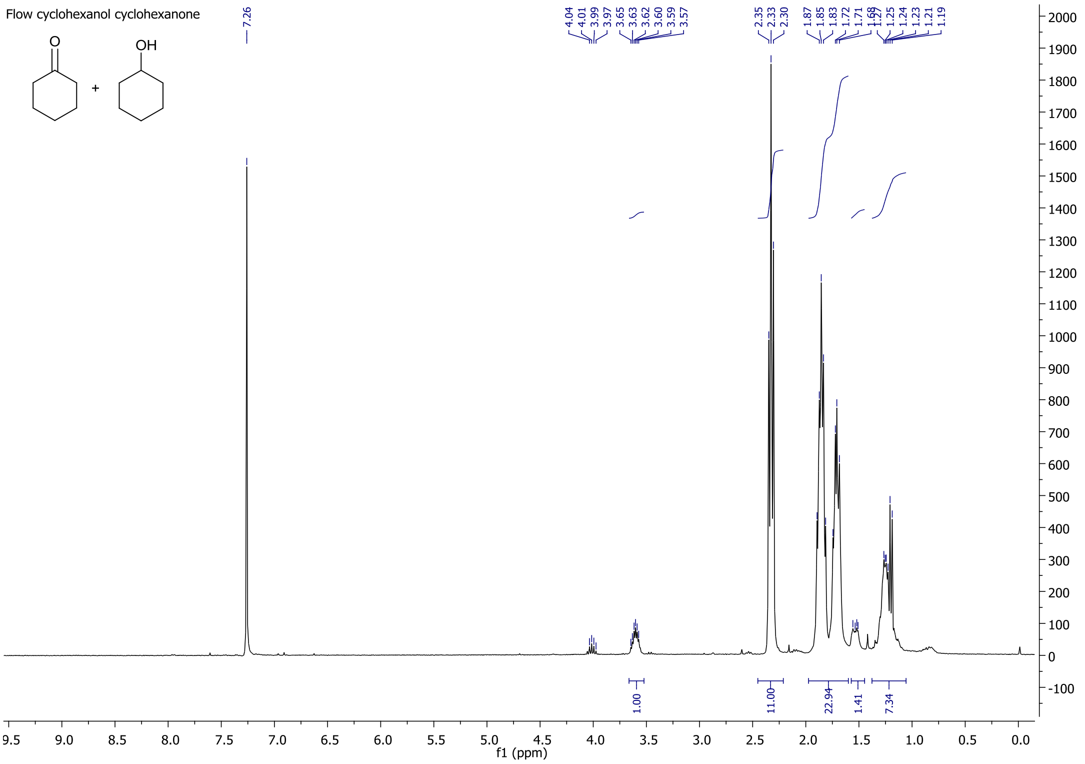


*Figure S33: $^{1}$H NMR spectrum of cyclohexanol and cyclohexanone.*

# 3-Heptanone (no 3-heptanol formation)

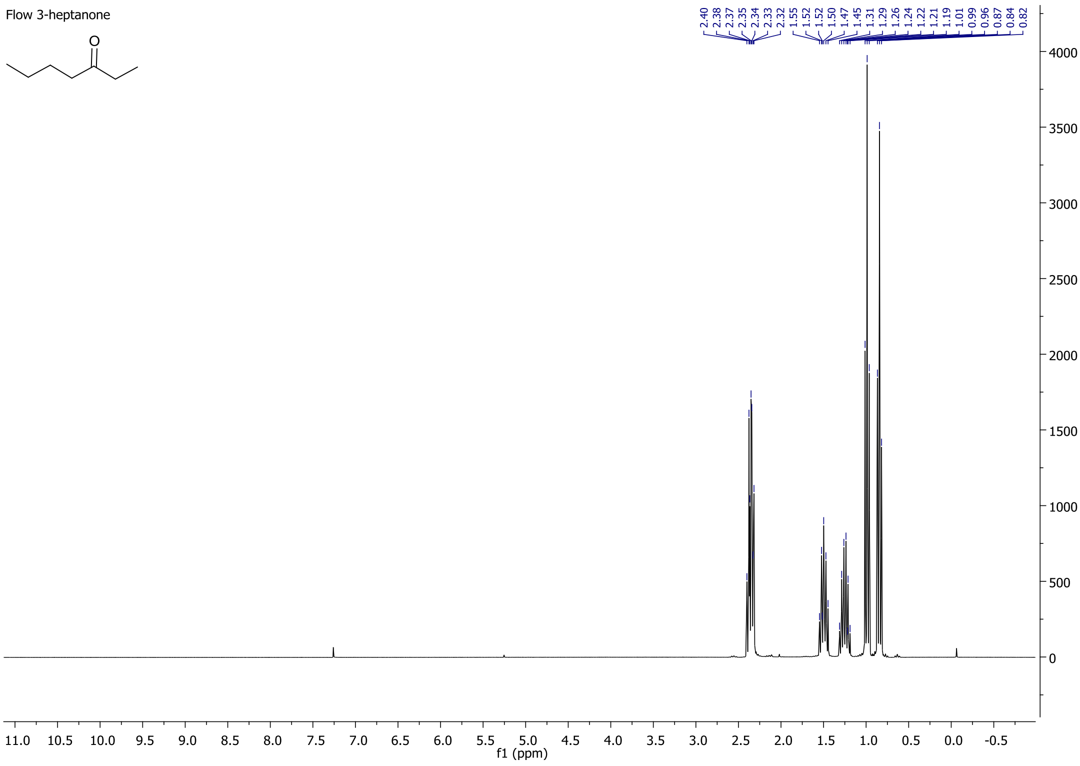


*Figure S34: $^1$H NMR spectrum of 3-heptanone.*

# 1-Phenylethanol and acetophenone mixture

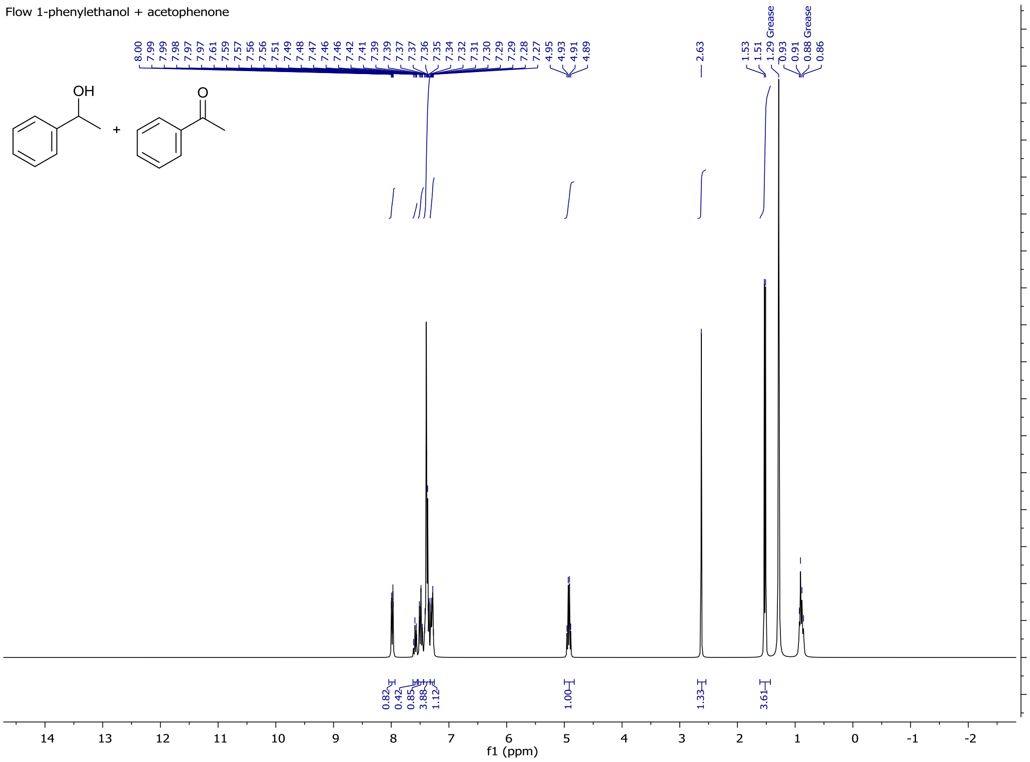


*Figure S35: $^1$H NMR spectrum of 1-phenylacetophenone and acetophenone.*

$^{1}$H NMR and $^{13}$C NMR spectrum of reduced benzaldehyde in the presence of a ketone: Benzaldehyde and acetophenone mixture

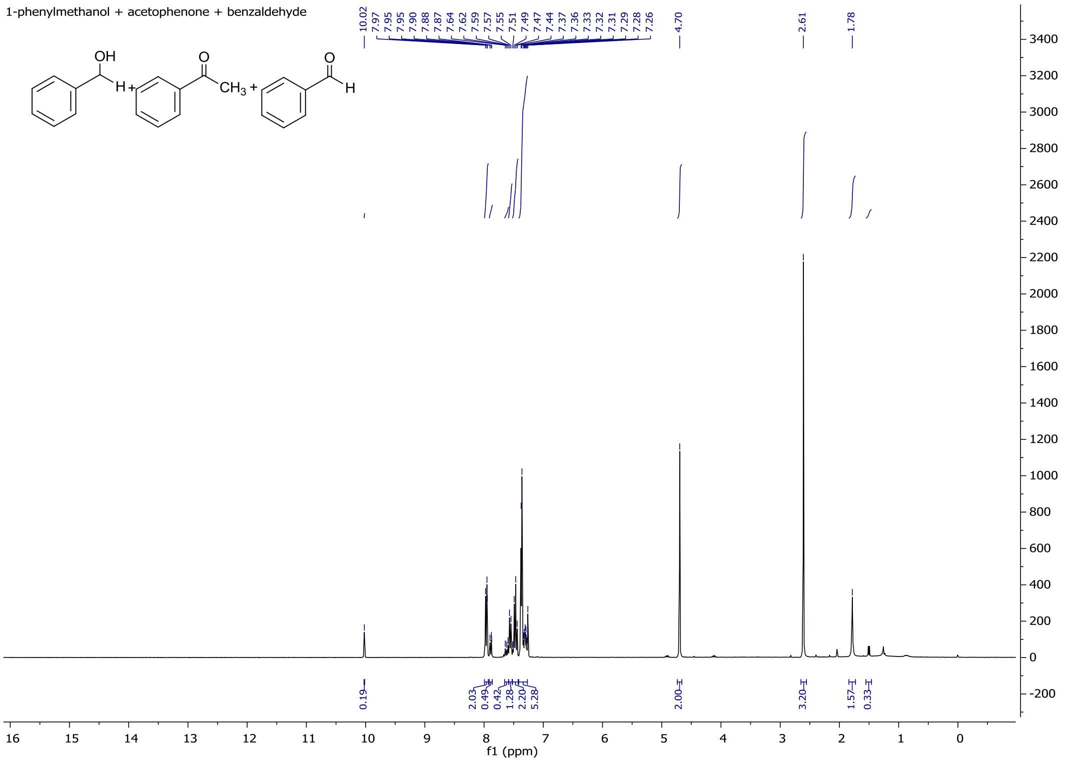


*Figure S36: $^{1}$H NMR spectrum of benzaldehyde, acetophenone and phenylmethanol mixture.*

Benzaldehyde and 3-heptanone mixture

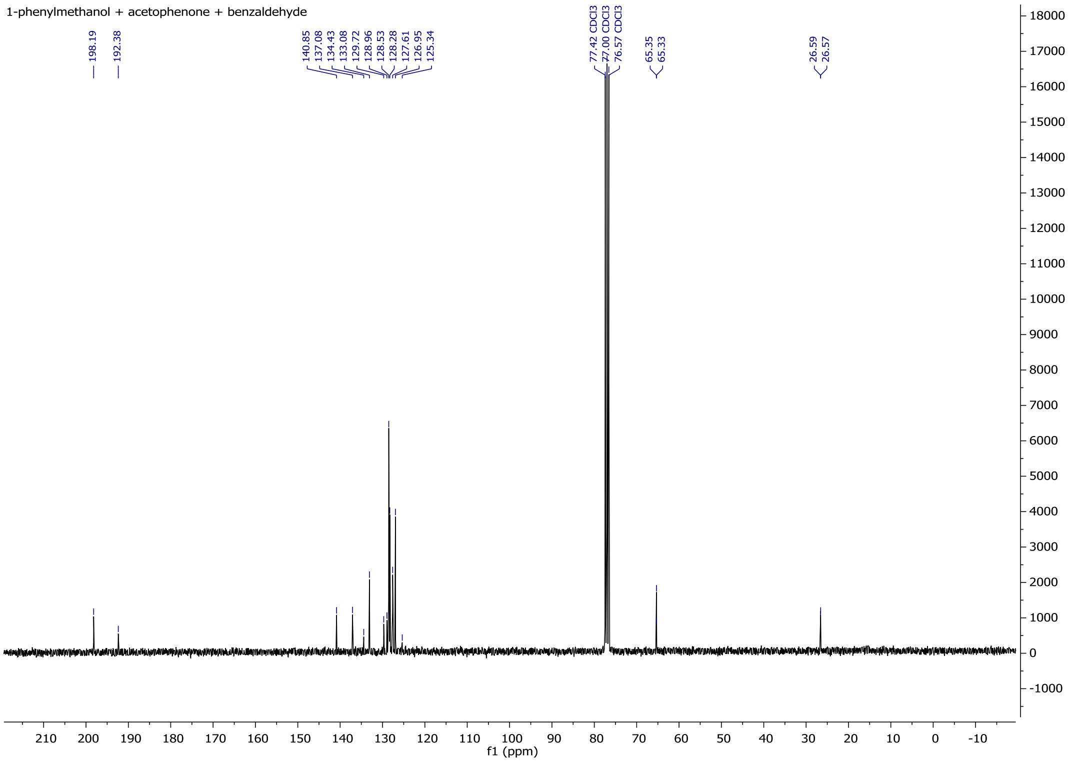


*Figure S37: $^{13}$C NMR spectrum of benzaldehyde, acetophenone and phenylmethanol mixture.*

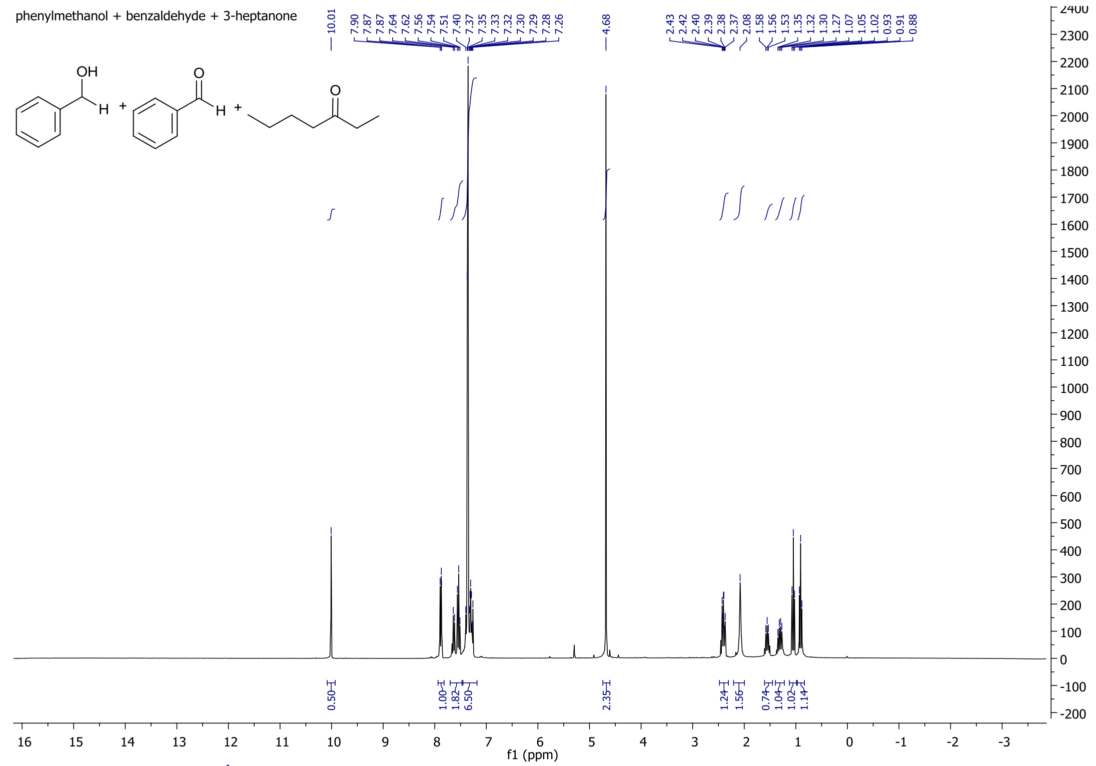


*Figure S38: $^{1}$H NMR spectrum of benzaldehyde, 3-heptanone and phenylmethanol mixture.*

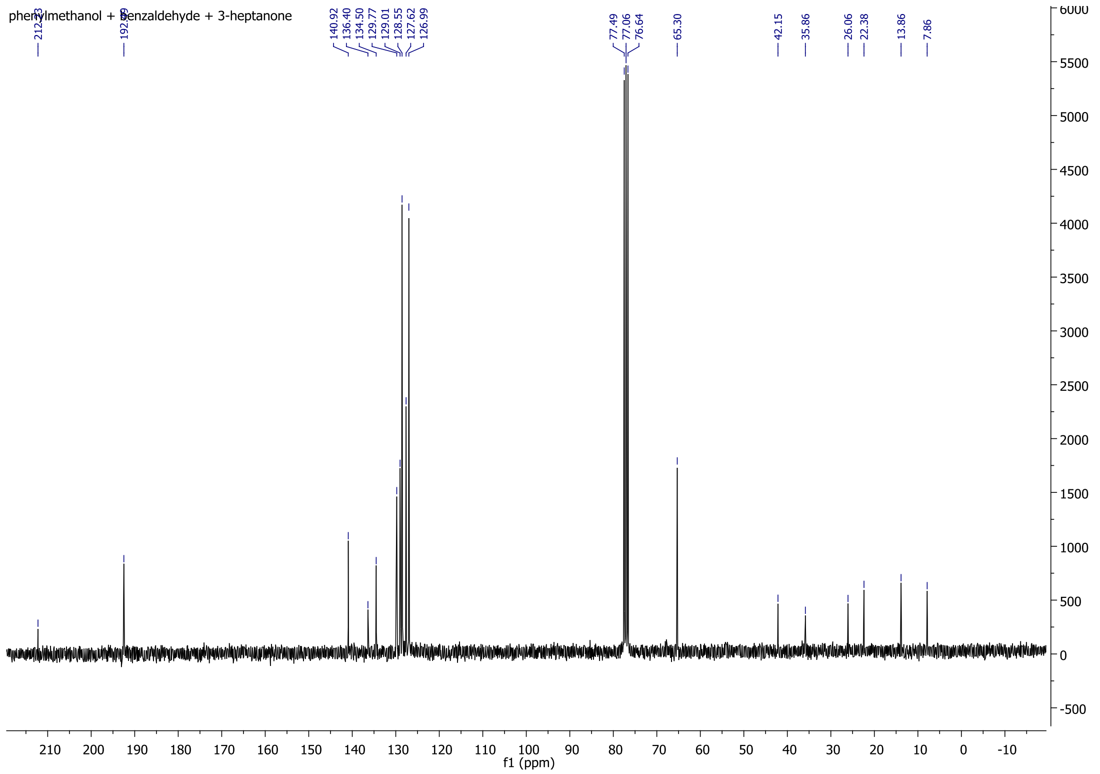


*Figure S39: $^{13}$C NMR spectrum of benzaldehyde, 3-heptanone and phenylmethanol mixture.*

## 3-Acetylbenzaldehyde and 1-(3-(hydroxymethyl)phenyl)ethenone mixture

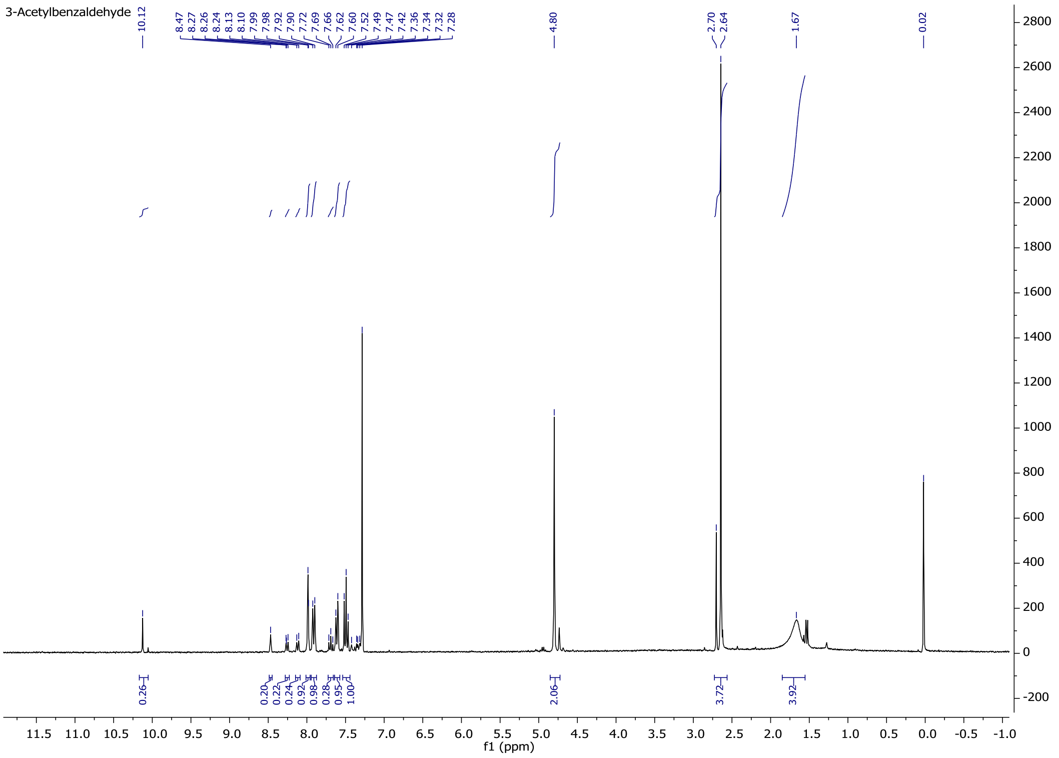


*Figure S43: $^{1}$H NMR spectrum of 3-Acetylbenzaldehyde and 1-(3-(hydroxymethyl)phenyl)ethenone mixture*
